# Small-molecule dissolution of stress granules by redox modulation benefits ALS models

**DOI:** 10.1038/s41589-025-01893-5

**Published:** 2025-05-14

**Authors:** Hiroyuki Uechi, Sindhuja Sridharan, Jik Nijssen, Jessica Bilstein, Juan M. Iglesias-Artola, Satoshi Kishigami, Virginia Casablancas-Antras, Ina Poser, Eduardo J. Martinez, Edgar Boczek, Michael Wagner, Nadine Tomschke, António M. de Jesus Domingues, Arun Pal, Thom Doeleman, Sukhleen Kour, Eric Nathaniel Anderson, Frank Stein, Hyun O. Lee, Xiaojie Zhang, Anatol W. Fritsch, Marcus Jahnel, Julius Fürsch, Anastasia C. Murthy, Simon Alberti, Marc Bickle, Nicolas L. Fawzi, André Nadler, Della C. David, Udai B. Pandey, Andreas Hermann, Florian Stengel, Benjamin G. Davis, Andrew J. Baldwin, Mikhail M. Savitski, Anthony A. Hyman, Richard J. Wheeler

**Affiliations:** 1 Max Planck Institute of Molecular Cell Biology and Genetics, Dresden, Germany; 2 Genome Biology Unit, European Molecular Biology Laboratory, Heidelberg, Germany; 3 Department of Chemistry, University of Oxford, Oxford, UK; 4 Kavli Institute for Nanoscience Discovery, University of Oxford, Oxford, UK; 5 Dewpoint Therapeutics, Boston, MA USA; 6 Department of Neurology, Technische Universität Dresden, Dresden, Germany; 7 Division of Child Neurology, Department of Pediatrics, Children’s Hospital of Pittsburgh, University of Pittsburgh Medical Center, Pittsburgh, PA USA; 8 Department of Human Genetics, University of Pittsburgh Graduate School of Public Health, Pittsburgh, PA USA; 9 Department of Neurology, University of Pittsburgh School of Medicine, Pittsburgh, PA USA; 10 Proteomics Core Facility, European Molecular Biology Laboratory (EMBL), Heidelberg, Germany; 11 Cluster of Excellence Physics of Life, TU Dresden, Dresden, Germany; 12 Biotechnology Center (BIOTEC), CMCB, TU Dresden, Dresden, Germany; 13 University of Konstanz, Department of Biology, Konstanz, Germany; 14 Konstanz Research School Chemical Biology, University of Konstanz, Konstanz, Germany; 15 Graduate Program in Molecular Biology, Cell Biology, and Biochemistry, Brown University, Providence, RI USA; 16 Department of Molecular Biology, Cell Biology, and Biochemistry, Brown University, Providence, RI USA; 17 German Centre for Neurodegenerative Diseases, Tübingen, Germany; 18 Center for Regenerative Therapies TU Dresden (CRTD), Technische Universität Dresden, Dresden, Germany; 19 Center for Transdisciplinary Neurosciences Rostock (CTNR), University Medical Center Rostock, University of Rostock, Rostock, Germany; 20 Translational Neurodegeneration Section ‘Albrecht Kossel’, Department of Neurology, University Medical Center Rostock, Rostock, Germany; 21 Deutsches Zentrum für Neurodegenerative (DZNE) Rostock/Greifswald, Rostock, Germany; 22 Department of Pharmacology, University of Oxford, Oxford, UK; 23 The Rosalind Franklin Institute, Harwell, UK; 24 Institute of Immunology and Infection Research, School of Biological Sciences, University of Edinburgh, Ashworth Laboratories, Edinburgh, UK; 25 Peter Medawar Building for Pathogen Research, Nuffield Department of Medicine, University of Oxford, Oxford, UK; 26 Present Address: Frontier Research Institute for Interdisciplinary Sciences, Tohoku University, Sendai, Japan; 27 Present Address: Blizard Institute, Barts and the London School of Medicine and Dentistry, Queen Mary University of London, London, UK; 28 Present Address: Faculty of Medicine, University Hospital Carl Gustav Carus, Technische Universität Dresden, Dresden, Germany; 29 Present Address: Dewpoint Therapeutics, Dresden, Germany; 30 Present Address: Dresden High Magnetic Field Laboratory (HLD), Helmholtz-Zentrum Dresden-Rossendorf (HZDR), Dresden, Germany; 31 Present Address: Department of Biochemistry, University of Toronto, Toronto, Ontario Canada; 32 Present Address: iHuman Institute, School of Life Science and Technology, ShanghaiTech University, Shanghai, China; 33 Present Address: Institute for Translational Bioengineering, pRED, Roche, Basel, Switzerland; 34 Present Address: Babraham Institute, Cambridge, UK

**Keywords:** Cell signalling, Neurodegenerative diseases, Target identification, Screening

## Abstract

Neurodegenerative diseases, such as amyotrophic lateral sclerosis, are often associated with mutations in stress granule proteins. Aberrant stress granule condensate formation is associated with disease, making it a potential target for pharmacological intervention. Here, we identified lipoamide, a small molecule that specifically prevents cytoplasmic condensation of stress granule proteins. Thermal proteome profiling showed that lipoamide stabilizes intrinsically disordered domain-containing proteins, including SRSF1 and SFPQ, which are stress granule proteins necessary for lipoamide activity. SFPQ has redox-state-specific condensate dissolving behavior, which is modulated by the redox-active lipoamide dithiolane ring. In animals, lipoamide ameliorates aging-associated aggregation of a stress granule reporter protein, improves neuronal morphology and recovers motor defects caused by amyotrophic lateral sclerosis-associated FUS and TDP-43 mutants. Thus, lipoamide is a well-tolerated small-molecule modulator of stress granule condensation, and dissection of its molecular mechanism identified a cellular pathway for redox regulation of stress granule formation.

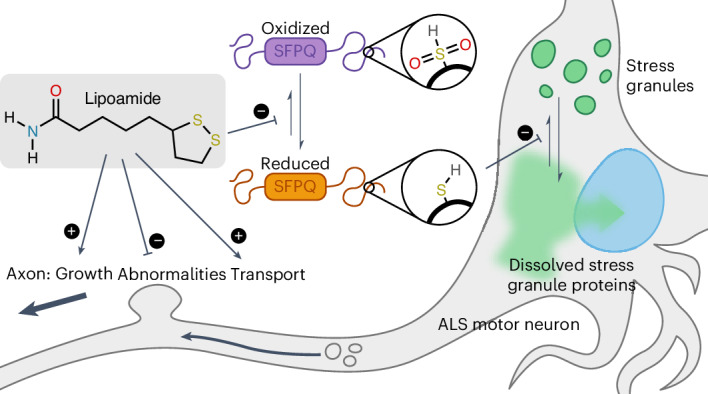

## Abstract (web summary)

Uechi et al. found that a small-molecule lipoamide dissolves stress granules (SGs) by targeting SFPQ, a redox-sensitive disordered SG protein, alleviating pathological phenotypes caused by amyotrophic lateral sclerosis-associated FUS and TDP-43 mutants.

## Main

Amyotrophic lateral sclerosis (ALS) is a fatal neurodegenerative disease that primarily affects motor neurons (MNs) with poor prognosis and few options for therapy^1^. Currently two Food and Drug Administration-approved small-molecule drugs are available on the market: riluzole and edaravone; the antisense oligonucleotide tofersen was also recently approved^2–4^. However, none block disease progression, and thus investigating new therapeutic routes is important to overcome ALS. Many mutations associated with familial ALS are found in RNA-binding proteins, notably, TAR DNA-binding protein 43 (TDP-43; also called TARDBP) and fused in sarcoma (FUS), with >40 ALS-associated mutations described in each^5–7^. These RNA-binding proteins have large intrinsically disordered regions (IDRs) with low sequence complexity.

TDP-43 and FUS are examples of stress granule proteins that normally localize to the nucleus, where they have crucial functions in gene expression regulation and DNA damage responses. For example, FUS localizes to paraspeckles and DNA damage foci in the nucleus^8,9^. Following cellular stress, TDP-43 and FUS are exported to the cytoplasm where they become incorporated into stress granules, although neither are necessary for stress granule formation^10^. Stress granules are liquid-like cytoplasmic assemblies, called condensates, that are formed by liquid–liquid phase separation of both nuclear exported and constitutively cytoplasmic proteins along with mRNA^11–13^. Stress granule formation is triggered by cell stress, such as oxidative stress. This is often dependent on the cytoplasmic stress granule protein G3BP1 (ref. ^10^). When cell stress is alleviated, stress granules dissolve, and proteins that normally reside in the nucleus, including FUS and TDP-43, translocate back to the nucleus.

It has been proposed that ALS-linked FUS and TDP-43 mutants cause diseases in part by inducing aberrant phase transitions of stress granules^12,14,15^. This reduces the dynamics of stress granule proteins, prevents them from dissolving when stress is removed and thus traps nuclear proteins in the cytoplasm. Supportive evidence is that FUS and TDP-43 mutants often show constitutive mislocalization to the cytoplasm, and FUS tends to aggregate more readily in the cytoplasm^16^. Both mechanisms may cause a loss-of-function phenotype in the nucleus or a gain-of-function (cytotoxic) phenotype in the cytoplasm as cytoplasmic aggregates or fibrils; these are associated with MN dysfunction leading to neurodegenerative disease^17–19^. Either way, (1) dissolving aberrant stress granules, (2) reducing sensitivity to triggers of stress granule formation, (3) preventing or reversing stress granule protein aggregation and/or (4) driving proteins back to the nucleus might be efficient ways to prevent or reverse the consequences of ALS. Indeed, compounds have been identified that can disrupt stress granule condensation, especially 1,6-hexanediol^20^ and similar alcohols^21^. However, these compounds are both toxic and nonspecific, as they affect multiple condensates^21,22^.

There is an unmet need for nontoxic and specific compounds that can dissolve specific condensates while leaving others unaffected. Here, we searched for such compounds targeting stress granules. We identify lipoamide as a tool compound to study stress granule biology and disease relevance. Such small molecules have been an essential tool for relating cytology to function. Lipoamide partitions into stress granules in cells, prevents formation of stress granules and dissolves existing stress granules, allowing us to identify a pathway that allows stress granules to sense the oxidative state of the cell. Interestingly, lipoamide alleviates pathology caused by ALS-associated FUS and TDP-43 mutants in both MNs in vitro and in fly models of ALS.

## Results

**Extended Data Fig. 1:**
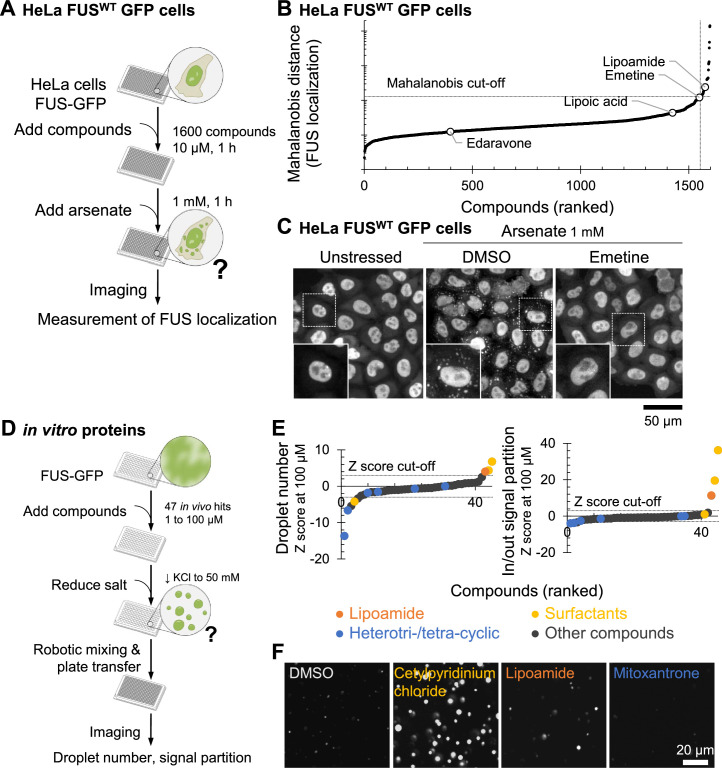
HeLa cell and *in vitro* follow-up screen identifying lipoamide. **a**, Workflow for screening small molecules for effects on FUS–GFP localization in HeLa cells *ex vivo*. **b**, Ranked Mahalanobis distances for all 1600 compounds screened (mean from six fields of view) where high values indicate more compound effect. Several automated measures of FUS localization were combined into a single Mahalanobis distance score; the largest contributors were cytoplasmic FUS condensate number and area (see the method section). A cut-off of 130 was used to select 47 compounds for further analysis. **c**, The subcellular localization of FUS–GFP in unstressed HeLa cells, stressed cells with compound solvent (DMSO) negative control, and with the positive control emetine, as observed in the screen in B. Stress causes nuclear export of FUS and formation of stress granules (cytoplasmic liquid FUS-containing condensates). Insets, magnified images in the boxed areas. **d**, Workflow for screening small molecules for effects on FUS condensation of purified FUS–GFP *in vitro*. **e**, Ranked Z scores of change in condensate droplet number and signal partition into FUS–GFP droplets (formed under low salt conditions) where larger positive or negative values mean more compound effect. Scores were calculated at the maximum concentration at which the compound solvent (DMSO) negative control had no significant effect; 100 μM. Lipoamide, surfactant and heterotri-/tetracyclic compounds are indicated by data point colour. **f**, Appearance of the droplets with compound solvent (DMSO) negative control or examples of compound classes: cetylpyridinium chloride (surfactant), lipoamide or mitoxantrone (heterotricyclic). Note the larger drops with cetylpyridinium chloride and lipoamide and the fewer smaller drops with mitoxantrone. Source data

We performed a cell-based screen of 1,600 small molecules from the Pharmakon library to identify compounds that affect stress granule formation following arsenate treatment by multiparameter automated image analysis of green fluorescent protein (GFP)-tagged FUS (FUS–GFP) localization in HeLa cells (Extended Data Fig. 1a). Many compounds altered FUS–GFP localization in stressed cells, often reducing the number of FUS–GFP-containing stress granules (Extended Data Fig. 1b). Emetine, known to prevent stress granule formation by stabilizing polysomes^23^, was present in the library and acted as a positive control (Extended Data Fig. 1c). Edaravone, a Food and Drug Administration-approved ALS therapeutic^24^, had no significant effect (Extended Data Fig. 1b). Compound classes that tended to have a large effect on FUS localization included cardiac glycosides, heterotri- and tetracyclic compounds (anthraquinones and acridines), surfactants and benzimidazoles.

The 47 strongest hits in HeLa cells were further tested in vitro for an effect on condensation of purified FUS–GFP under physiological (low-salt (50 mM KCl) and reducing (1 mM DTT)) conditions, with the aim of selecting for compounds that can directly affect stress granule proteins (Extended Data Fig. 1d). Seven compounds significantly affected FUS–GFP condensates in vitro and fell into three compound classes (Extended Data Fig. 1e,f). Of these, surfactants have no plausibility as a systemic therapeutic, and heterotri- and tetracyclic compounds have previously been investigated for antiprion properties with limited success^25^. Lipoamide was a novel hit for stress granule modulation. Lipoic acid, a related compound featuring a carboxylic acid instead of a carboxamide, was a weaker hit in HeLa cells with a qualitatively similar effect on the number of stress granules.

### Lipoamide reverses stress granule formation in culture

**Fig. 1:**
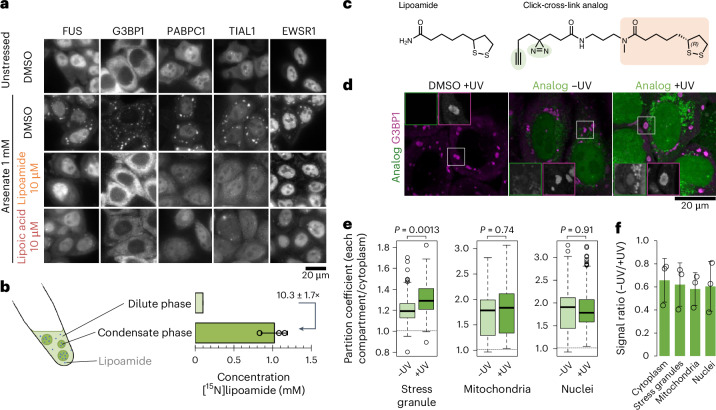
Lipoamide reduces cytoplasmic condensation of stress granule proteins by partitioning into condensates. **a**, Representative images of HeLa cells expressing GFP-tagged stress granule markers (G3BP1, PABPC1, TIAL1 or EWSR1) from three independent experiments. Cells were pretreated with 10 μM lipoamide or lipoic acid (with DMSO solvent control) for 1 h, followed by 1 mM arsenate for 1 h or DMSO without arsenate. **b**, Left, schema of lipoamide partitioning into FUS condensates in vitro. Right, mean ± s.e.m. of the concentration of racemic [^15^N]lipoamide in the condensate and the surrounding dilute phase of FUS–GFP in vitro, quantified using ^15^N(^1^H) NMR from four independent experiments. **c**, Chemical structure of the click-cross-link lipoamide analog, with the lipoamide backbone (orange) and the groups for UV cross-linking and click reaction (green). **d**, Representative images from three experiments using HeLa cells treated with 3 mM arsenate for 1 h, followed by 30 µM analog or control DMSO in the presence of arsenate for an additional 30 min before either irradiation with UV for cross-linking (+UV) or not (–UV), followed by fixation, immunostaining and click reaction with the fluorophore. Stress granules were labeled with G3BP1. Insets, stress granules in the boxed areas (analog and G3BP1 are boxed in green and magenta, respectively). **e**, Box plot of the partition coefficient of the analog into stress granules, mitochondria or nuclei relative to the cytoplasm (excluding stress granules and mitochondria) based on signal intensity of the fluorophore. Box plots show the median (bold bar), 25th and 75th percentiles and outliers (open dots), and whiskers extend to the most extreme values; *n* = 344 (–UV) and 345 (+UV) cells from three experiments. *P* values were determined by unpaired two-tailed *t*-test. **f**, Mean ± s.d. of signal intensity ratio (–UV against +UV) of the fluorophore in the indicated subcellular compartments from *n* = 3 experiment replicates. Source data

**Extended Data Fig. 2:**
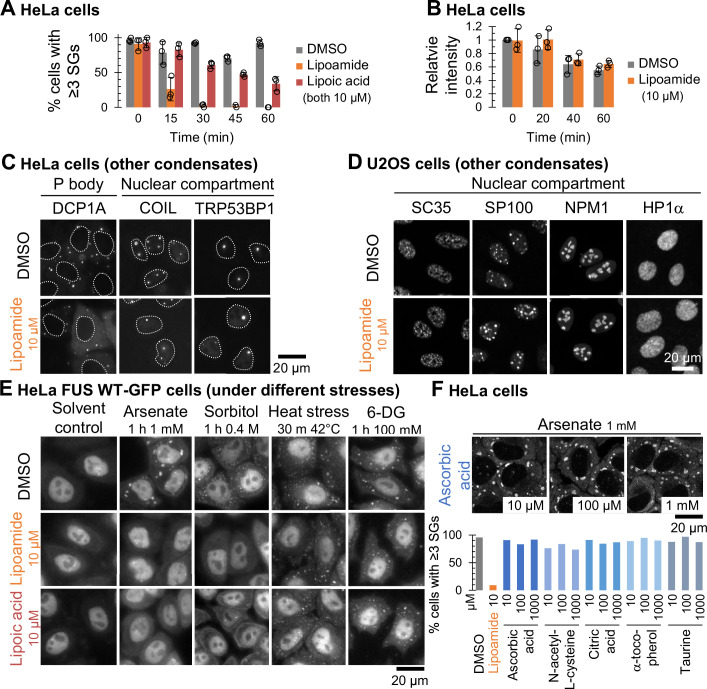
Lipoamide characterization in HeLa cells. **a**, Mean ± s.d. of percentage of HeLa cells with ≥ 3 G3BP1-positive stress granules (SGs). Cells were treated with 1 mM arsenate for 1 h to induce SGs, followed by 10 µM lipoamide, lipoic acid, or 0.1% DMSO (control) in the presence of arsenate for indicated minutes. *n* = 52–248 cells from 3 independent experiments. **b**, Mean ± s.d. of relative intensity levels of puromycin normalized to those of GAPDH in HeLa cells treated with 10 µM lipoamide (0.1% DMSO as the control) for 1 h, followed by 1 mM arsenate for 1 h and 91.8 µM puromycin for 5 min, detected by immunoblotting from 3 independent experiments. **c**, Images of HeLa cells expressing GFP-tagged markers of other membrane-less organelle compartments subjected to 1 h treatment with 10 μM lipoamide (or DMSO control). Where unclear, thep position of nuclei is indicated with a broken outline. Lipoamide does not disrupt P bodies (DCP1A), Cajal bodies (COIL), or DNA damage foci (TRP53BP1). **d**, Images of U2OS cells stained with indicated markers of other membrane-less subcellular compartments subjected similarly to HeLa cells in G. Lipoamide also did not disrupt nuclear speckles (SC35), PML bodies (SP100), nucleoli (NPM1), or heterochromatin (HP1α). **e**, Images of HeLa cells expressing FUS–GFP subjected to different stresses – arsenate, sorbitol (osmotic), heat, or 6-deoxyglucose (DG; glycolysis) – with concurrent treatment with 10 μM lipoamide or lipoic acid. **f**, HeLa cells were treated with 0.1% DMSO (control), 10 µM lipoamide, or indicated concentrations of other known and potential antioxidants (ascorbic acid as representative images on the top) for 1 h, followed by 1 mM arsenate for 1 h in the presence of each chemical. SGs were labeled with G3BP1. Bottom, percentage of HeLa cells with ≥ 3 G3BP1-positive SGs from *n* = 119–202 cells. Source data

To test whether lipoamide and lipoic acid affect stress granule formation or solely partitioning of FUS into stress granules, we treated five HeLa cell lines that express different GFP-tagged stress granule proteins with new stocks of lipoamide or lipoic acid. Pretreatment with either compound for 1 h before 1 h of arsenate stress prevented cytoplasmic condensation for all proteins we tested, including G3BP1 (Fig. 1a). The addition of lipoamide and lipoic acid to arsenate-stressed cells, in the continued presence of arsenate, also led to dissolution of pre-existing stress granules (Extended Data Fig. 2a). In arsenate-stressed cells, protein synthesis was impeded. Using a puromycin incorporation assay, we showed that protein translation does not significantly differ between lipoamide-pretreated and control arsenate-stressed cells (Extended Data Fig. 2b).

To assess whether lipoamide acts specifically on stress granules, we tested its effects on nine other intracellular condensates (three cytoplasmic and six nuclear) and found that these other condensates were not affected (Extended Data Fig. 2c,d and Extended Data Table 1). The specificity extended to stressor types, as stress granule formation induced by oxidative stress and osmotic shock was inhibited, whereas stress granules still formed in the presence of lipoamide after heat treatment or inhibition of glycolysis (Extended Data Fig. 2e). Therefore, we conclude that lipoamide activity is comparatively specific regarding modulation of cellular condensates.

**Extended Data Fig. 3:**
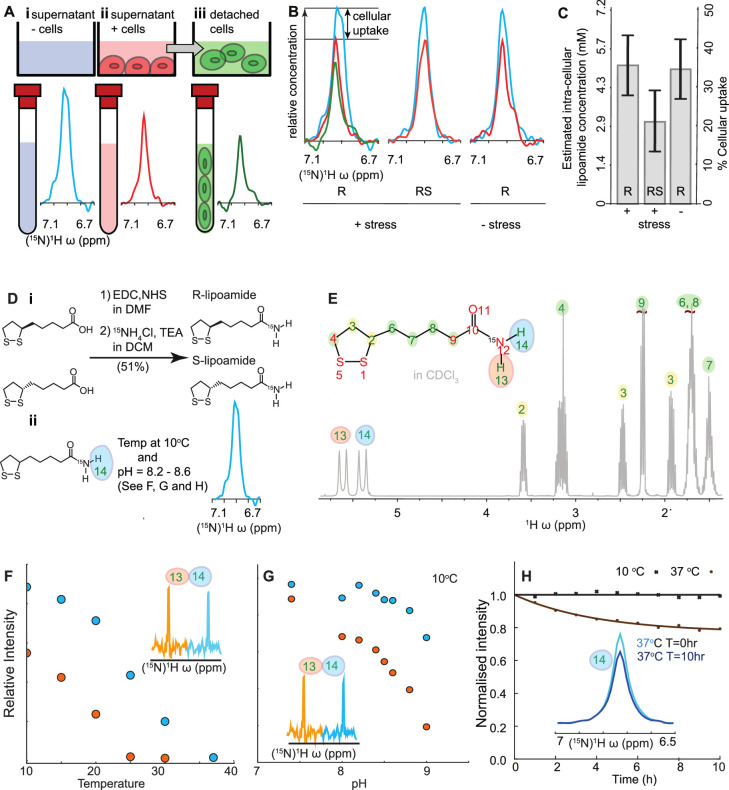
Tracking cellular uptake of [^15^N]-Lipoamide using NMR. **a**, Methodology for quantitation of [^15^N]-lipoamide uptake by HeLa cells, using the trans-amide proton to measure [^15^N]-lipoamide concentration (see F- H). Medium with 100 μM [^15^N]-lipoamide was incubated for 1 h in the absence or presence of HeLa cells. Following removal of medium, the cells were washed with medium (without arsenate) and detached using EDTA-trypsin. Solution or cell pellet/in-cell NMR was used to determine [^15^N]-lipoamide concentration. Example spectra for cells stressed with 3 mM arsenate and incubated with R-(+)-lipoamide are shown with the same y axis scale. **b**, Cellular uptake was determined by subtracting signal from medium incubated with cells (red) from signal from medium without cells (blue). This was carried out for all four combinations of stressed (3 mM arsenate) or unstressed cells with [^15^N]-(*R*)-(+) or (±)-lipoamide. For stressed cells treated with [^15^N]-(*R*)-(+)-lipoamide the high signal intensity from the washed cell sample (green) is consistent with the large uptake from the medium calculated from the with (red) and without cell (blue) signal intensity. **c**, Quantitation of B showing percentage uptake and calculated intracellular concentration, assuming that lipoamide is uniformly distributed within cells (see Supplemental Methods). Uncertainty in measurement was approximately 30% and there was no significant difference in uptake between conditions. All measurements indicated substantial uptake of lipoamide and cellular concentrations >1 mM. **d**, Overview of synthesis of [^15^N]-lipoamide, highlighting the trans amide proton (14). **e**, ^1^H NMR spectrum of [^15^N]-lipoamide in CDCl_3_. Peaks can be unambiguously assigned to individual proton environments. **f-h**, Controls determining reliability of quantitation of [^15^N]-lipoamide using the amide protons in ^15^N edited ^1^H NMR experiments. **f**, Dependency of the cis (13) and trans (14) amide proton signal on temperature, at a constant pH of 8.3. Both resonances decreased with increasing temperature, indicating local molecular dynamics and/or interactions with H_2_O on ms to µs timescale reduce the signal. Trans amide proton resonance approaches a plateau towards 10 °C. **g**, Dependency of the cis and trans amide proton signal on pH, at a constant temperature of 10 °C. Together, indicating at 10 °C and below pH 8.6 integrated signal intensity of the trans-amide proton of lipoamide in ^15^N edited ^1^H NMR experiments is a reliable proxy for concentration. **h**, Signal intensity of the trans-amide proton of lipoamide, when dissolved in growth medium, decreased over time at 37 °C but not at 10 °C. At 10 °C signal intensity is stable for >10 h experiments. Source data

To confirm that lipoamide enters cells and to determine its intracellular concentration, we synthesized [^15^N]lipoamide, which can be quantitatively detected by ^15^N(^1^H) NMR (Supplementary Note 1 and Extended Data Fig. 3). Following treatment of HeLa cells with 100 µM [^15^N]lipoamide, loss of [^15^N]lipoamide signal from the growth medium indicated that it accumulates in millimolar concentrations in cells. There was also a corresponding gain in [^15^N]lipoamide signal in the cell pellet (Extended Data Fig. 3a–c). These findings indicate that lipoamide accumulates at high concentrations in cells.

**Extended Data Fig. 4:**
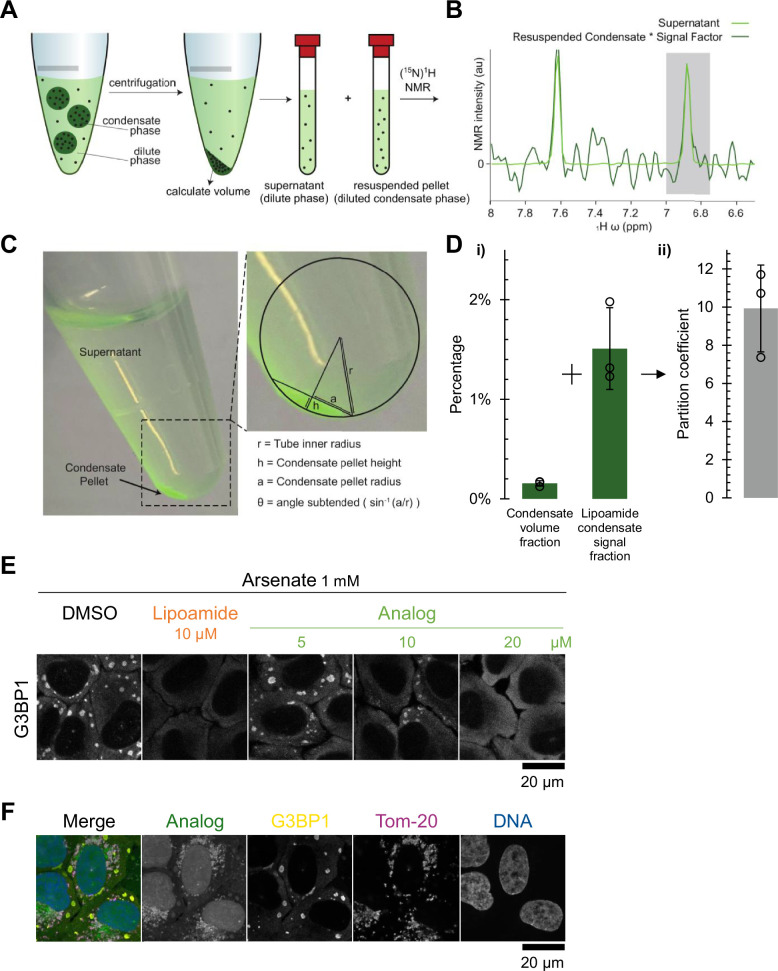
Experimental set up for portioning assays of lipoamide and its analog. **a-d**, Methodology for determination of partition of [^15^N]-lipoamide into FUS condensates *in vitro*. **a**, Schematic showing the sample preparation process. **b**, Example of ^15^N edited ^1^H NMR signal around the ^15^N cis and trans amide protons for a dilute phase and condensate phase. Condensate phase spectrum is shown scaled by an experimentally determined signal factor, used in calculation of the signal fraction. **c**, Measurements for calculation of condensate pellet volume from macro photographs of the sample within a microcentrifuge tube. **d**, Mean ± s.d. of measured ^15^N edited ^1^H NMR signal fraction and condensate volume fraction (from 4 independent experiments) and calculated partition coefficient. Alternative presentation of the data in Fig. 2a. **e**, Representative images of HeLa cells pre-treated with indicated concentrations of lipoamide or the click-crosslink lipoamide analog in Fig. 2b for 1 h followed by 1 mM of arsenate for additional 1 h in the presence of compounds. SGs were labeled with G3BP1. **f**, The images of HeLa cells treated with the analog and subjected to arsenate treatment and UV cross-linking from Fig. 2c, with a channel of Tom-20 as a mitochondrial marker. Source data

We used two strategies to ask whether lipoamide partitions into stress granules. First, we used [^15^N]lipoamide with FUS condensates as a minimal in vitro model of the stress granule environment using an abundant stress granule protein. [^15^N]Lipoamide from the dilute phase following FUS–GFP condensation under low-salt reducing conditions (Extended Data Fig. 4a–d) partitioned into the FUS–GFP condensate phase by a factor of ten (Fig. 1b). To analyze partitioning of lipoamide into stress granules in cells, we synthesized a lipoamide analog derivatized with a diazirine (for UV-induced cross-linking) and alkyne (for click chemistry) groups (Fig. 1c). This dissolved stress granules with a slightly lower potency than lipoamide (Extended Data Fig. 4e) but allowed us to cross-link this analog to proteins in the vicinity by UV irradiation, subsequently labeling it via click reaction with a fluorophore. Using this clickable cross-linking analog at 30 µM (insufficient to dissolve stress granules induced with 3 mM arsenate), we observed signal particularly in nuclei (labeled by DAPI), stress granules (labeled with anti-G3BP1) and mitochondria (labeled with anti-TOM20). Similar localization was observed both with and without UV-induced cross-linking, although UV cross-linking increased the relative signal in stress granules (Fig. 1d,e and Extended Data Fig. 4f). Comparison of signal intensity with and without cross-linking suggests that >50% of the lipoamide analog molecules have high-affinity interactions with fixable macromolecules (Fig. 1f). Together, these data indicate that lipoamide partitions into stress granules, along with other organelles.

### More potent lipoamide analogs require a ditholane ring

**Fig. 2:**
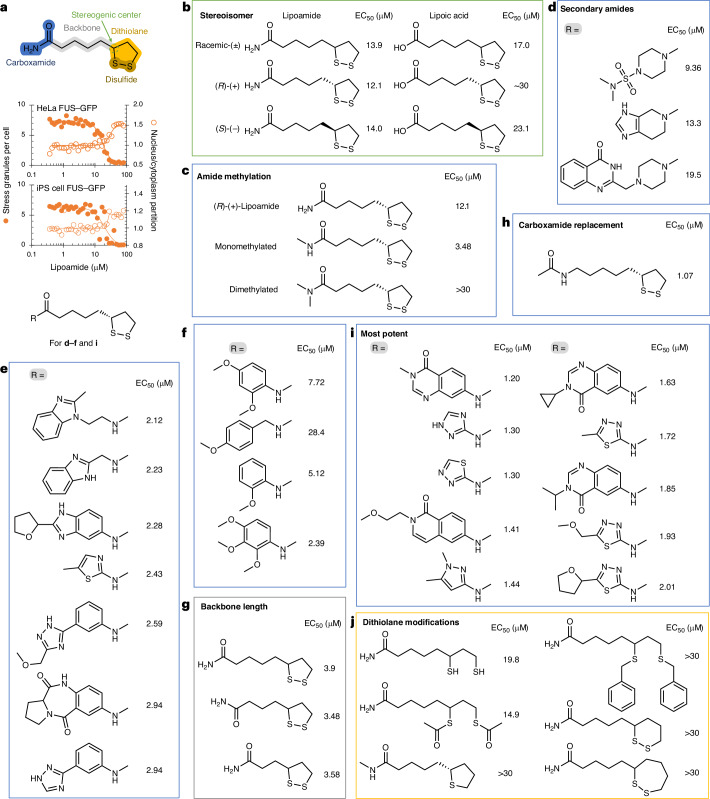
SAR shows that lipoamide activity is dependent on the dithiol but is nonenzymatic. **a**, Top, schema of the chemical structure of lipoamide (racemic), highlighting its features. Bottom, lipoamide dose–response using HeLa and iPS cells, showing FUS–GFP condensate (stress granule) number (solid circles, left axis) and nuclear/cytoplasmic signal ratio (open circles, right axis) with 1 h pretreatment with lipoamide followed by 1 h of arsenate stress under continued lipoamide treatment. **b**–**j**, Chemical structures and EC_50_ values of lipoamide and its derivatives using HeLa cells and the treatment scheme in **a**. EC_50_ values were calculated from dose–response curves (Methods), and each concentration of each compound was tested in duplicate wells (*n* = 1,750–2,650 cells per well) with two independent experiments. **b**, Enantiomers of lipoamide and lipoic acid. **c**, Comparison of mono- and dimethylated lipoamide. **d**–**f**, Additional carboxamide analogs of lipoamide. **g**, Modifications of the linker length between the carboxamide and the dithiolane ring of lipoamide. **h**, Substitution of the carboxamide of lipoamide. **i**, Carboxamide analogs of 6-amino-3-substituted-4-quinazolinones and five-membered aminoheterocyclic amides. **j**, Modifications of the dithiolane ring of lipoamide or similar compounds. Source data

To determine which chemical features of lipoamide are required for activity, we synthesized a panel of lipoamide-like compounds and tested the structure–activity relationship (SAR). As a reference, we confirmed lipoamide potency; lipoamide pretreatment, in both HeLa and induced pluripotent stem (iPS) cells, caused a dose-dependent change in two targeted measures of FUS–GFP localization: a decrease in stress granule numbers and an increase in partitioning of FUS–GFP back to the nucleus with a similar dose dependency (Fig. 2a). Titration analyses of the series of lipoamide analogs identified 16 compounds with more than approximately fivefold increased potency (half-maximum effective concentration (EC_50_) < 2.5 µM; Fig. 2b–j) compared to lipoamide. Specifically, lipoamide derivatives of 6-amino-3-substituted-4-quinazolinones and five-membered aminoheterocyclic amides mostly showed EC_50_ values below 2 µM (Fig. 2i).

The (*R*) and (*S*) isomers of lipoamide and lipoic acid had a similar EC_50_, indicating little stereoisomer specificity (Fig. 2b). The chemical structure of lipoamide is similar to that of the lipoyl moiety, used as a hydrogen-accepting cofactor by two mitochondrial Krebs cycle enzymes, which is recycled to the oxidized state by dihydrolipoamide dehydrogenase (DLD; also mitochondrial)^26^. Cells exclusively use the (*R*)-lipoyl moiety stereoisomer. However, the comparable EC_50_ between the (*R*) and (*S*) isomers of lipoamide and the absence of a lipoate ligase in eukaryotic cells^26^ indicate that lipoamide does not primarily function through these mitochondrial proteins to affect stress granule dissolution.

Monomethylation of lipoamide on the amide improved activity, whereas dimethylation reduced it (Fig. 2c). However, other disubstituted amide analogs showed activity in the context of more complex amide structures (Fig. 2d). Indeed, many monosubstitutions of the amide improved activity, with no clear trend for beneficial substitutions, that is, a relatively ‘flat’ SAR space in the carboxamide group. Dissimilar substitutions could similarly increase potency to the low micromolar range (Fig. 2e), whereas some similar heterocycle substitutions could have a wide range of potencies (Fig. 2f). Activity could be retained and even increased by shortening the alkane ‘backbone’ (Fig. 2g). A compound without a carboxamide or carboxylic acid moiety (that is, unlike both lipoamide and lipoic acid) was active, with the highest potency tested (Fig. 2h), although similarly and robustly potent compounds were monosubstituted amides (Fig. 2i).

Importantly, the dithiolane ring is necessary for activity, indicating a redox activity for stress granule dissolution. Lipoamide derivatives are likely reduced in the cellular environment. Indeed, the reduced dihydrolipoamide form was active (Fig. 2j). Furthermore, a labile thiol modification (two thioesters) was active, whereas nonlabile derivatives (thiol benzylation and substitution to a tetrahydrothiophene, a thiolane ring) were not (Fig. 2j). Six- and seven-membered disulfide rings removed activity (Fig. 2j). As redox potential and kinetics are linked to disulfide ring size^27,28^, this also indicated a redox-linked mechanism. Because edaravone and other known and potential redox-active compounds^29–31^ did not reduce stress granules at micromolar concentrations comparable to lipoamide (Extended Data Fig. 2f), this suggests that lipoamide has more redox potency than those compounds to control stress granule dynamics. Together, the SAR data suggest that lipoamide acts through a nonenzymatic route and likely through a redox-associated process.

### Lipoamide increases FUS condensate liquidity in vitro

**Extended Data Fig. 5:**
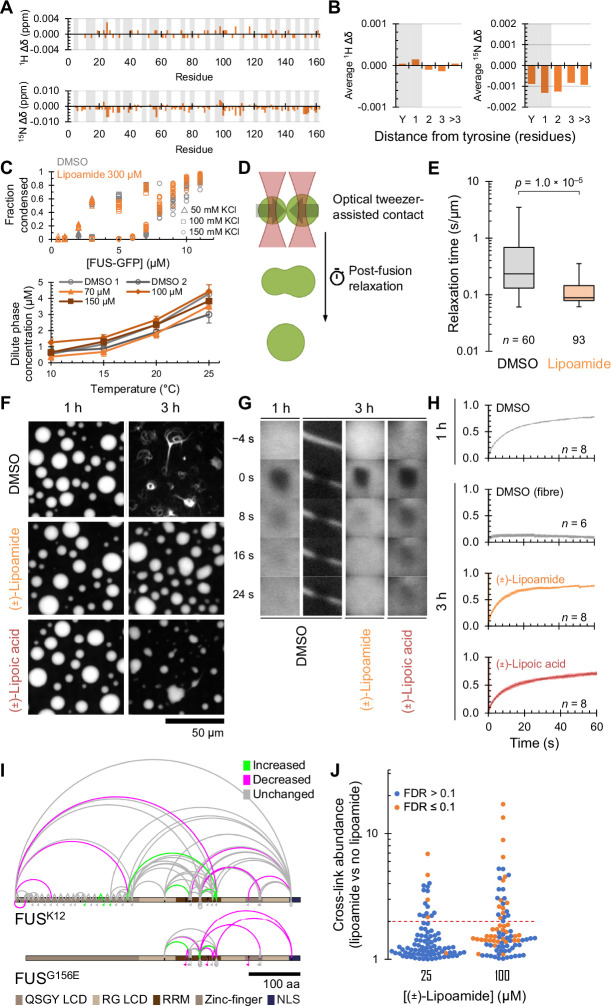
Lipoamide weakly increases liquidity of FUS condensates *in vitro.* **a**, NMR chemical shift deviations per residue for the FUS N-terminal PLD (residues 1 to 163) with 500 μM lipoamide compared to the drug solvent control (1% DMSO). Light gray bars indicate tyrosine residues and residues neighboring a tyrosine. **b**, Average ^1^H and ^15^N shifts across residues zero, one, two, three or more than three residues from a tyrosine in the presence of lipoamide. **c**, Top, fractions of FUS proteins condensed at indicated salt (KCl) concentrations in the presence of 300 µM lipoamide or the DMSO control (0.3% v/v). *n* = 16 image fields for each condition. Bottom, dilute phase concentrations (equivalent to saturation concentrations) of FUS–GFP at 150 mM KCl at different temperatures and lipoamide concentrations (errors are s.d.) **d**, Schematic illustrating the quantitation of condensate droplet liquidity using optical tweezers. Two droplets are brought into contact and begin to fuse: the time taken to relax to a single spherical droplet (once adjusted for the geometric mean radius as the characteristic droplet size) is a measure of the viscosity to surface tension ratio of the droplet – a proxy of liquidity. **e**, Droplet size-corrected relaxation times for droplet fusions with either 300 μM lipoamide (*n* = 93 independent fusion event) or equivalent DMSO solvent control (0.3%, *n* = 60). Box represents the 25^th^, 50^th^ and 75^th^ percentiles, whiskers represent 5^th^ and 95^th^ percentiles. *p* value by unpaired two-tailed *t*-test. Lipoamide reduces fusion time, indicating lower viscosity and/or greater surface tension. **f–h**, Effect of 30 μM lipoamide or lipoic acid on FUS G156E-GFP condensates ‘aging’, relative to an equivalent DMSO solvent control (0.3%). Condensates were formed under 50 mM of KCl while shaking. **f**, Representative images after 1 and 3 h aging, showing fiber formation in the DMSO sample in contrast to the lipoamide or lipoic acid samples. **g**, Representative fluorescence recovery after photobleaching (FRAP) time series of FUS condensates and fibers at corresponding time points. **h**, Mean ± s.d. of relative intensity of FUS–GFP FRAP in G. Aged (3 h) condensates treated with lipoamide or lipoic acid maintain large FUS-GFP mobile fraction. Both compounds delay fiber formation. **i**, Changes in intramolecular crosslinking due to lipoamide of FUS in *in vitro* low salt (80 mM KCl) condensates using the lysine-rich FUS K12 or FUS G156E. Significantly changed crosslinking sites with a change in intensity of more than two-fold and FDR ≤ 0.1; 3 independent experiments) are shown coloured in green (increased) or red (decreased). Other crosslinking sites are shown in gray. **j**, Dose-dependent effect of lipoamide on FUS K12, plotting absolute change in crosslink intensity relative to no lipoamide. Crosslinking sites with false discovery rate (FDR) > 0.1 are shown in blue, those with FDR ≤ 0.1 in orange (2 independent experiments). Two-fold change is indicated with a dashed red line. Source data

To test if lipoamide undergoes strong interactions with known stress granule proteins, we turned again to FUS using classical methods including isothermal titration calorimetry (ITC) and chemical shift perturbation in ‘fingerprint’ ^1^H-^15^N two-dimensional protein NMR spectra. We could not detect interactions between FUS–GFP and lipoamide in vitro by ITC. NMR of the N-terminal prion-like domain of FUS showed only extremely weak ^1^H and ^15^N shifts in the presence of lipoamide (Extended Data Fig. 5a,b). To test if lipoamide alters FUS condensate formation, we examined the critical salt concentration and temperature of in vitro FUS condensates but found no detectable change in the presence of lipoamide (Extended Data Fig. 5c).

We then tested whether lipoamide alters FUS condensate properties, first testing the effect on in vitro condensate liquidity using laser optical tweezers to assay droplet fusion. This showed significantly decreased droplet fusion times in the presence of lipoamide and thus increased liquidity (higher ratio of surface tension to viscosity; Extended Data Fig. 5d,e). Over time FUS condensates gradually harden, visible as an increasing viscosity and decreasing mobile fraction of FUS, and eventually form solid fibers. This is particularly prominent for condensates of the ALS-linked mutant FUS-G156E, which hardens and forms fibers rapidly^12^. We tested whether lipoamide maintains condensate liquidity using fluorescence recovery after photobleaching (FRAP). Both lipoamide and lipoic acid reduced FUS-G156E–GFP condensate hardening and fiber formation (Extended Data Fig. 5f–h).

Finally, we turned to mass spectrometry to analyze changes in FUS-G156E self-interaction in vitro in the presence of lipoamide. We used lysine–lysine chemical cross-linking and, following tryptic digest, mass spectrometry detection of the cross-linked peptides as evidence for inter- and intramolecular interactions under different conditions. This technique requires lysine residues, which the N-terminal IDR of wild-type (WT) FUS lacks. Therefore, we also analyzed a FUS mutant with 12 lysine substitutions in the N-terminal domain. Lipoamide caused a change, predominantly decrease, in the intensity of identified lysine–lysine cross-linking sites and therefore suggested reduced FUS–FUS interactions (Extended Data Fig. 5i,j). Together, lipoamide has a weak effect on FUS condensate properties in vitro by modulating FUS–FUS interactions and does so without strong small-molecule-protein binding typically detectable by ITC or NMR.

### Arginine/tyrosine-rich IDRs are stabilized by lipoamide

**Fig. 3:**
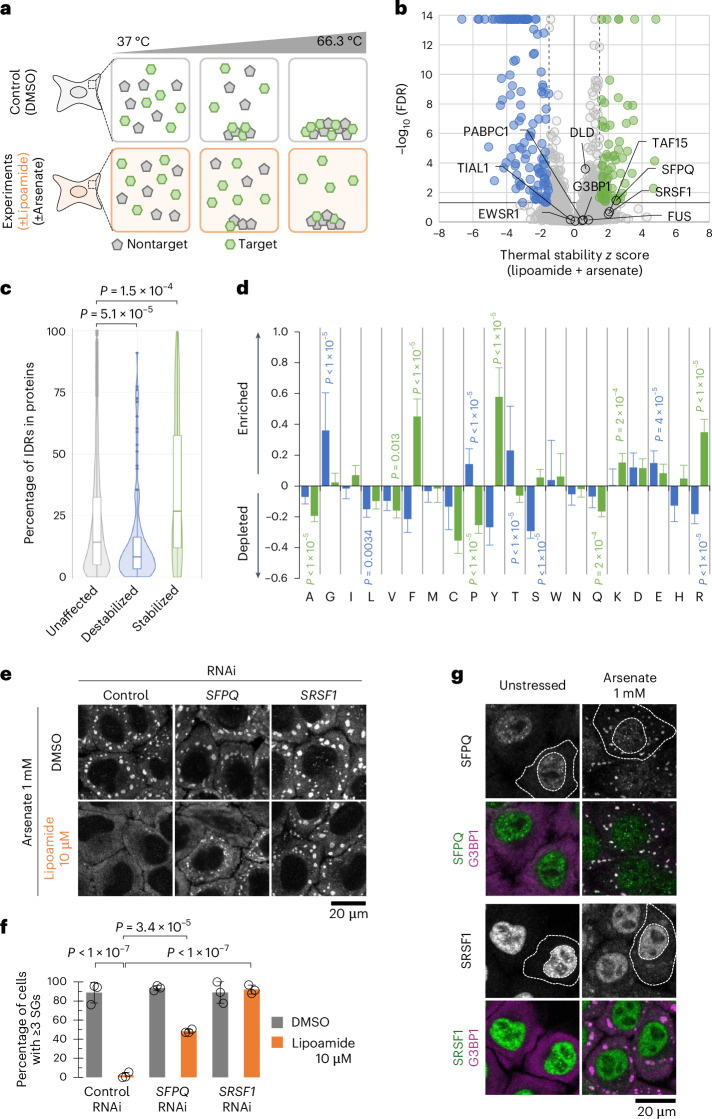
Lipoamide interacts with disordered proteins in cells. **a**, TPP scheme for testing the effect of lipoamide on protein thermal stability. Soluble protein quantity was measured by mass spectrometry, normalized to 0.1% DMSO-treated controls, of HeLa cells treated with 100 µM lipoamide and/or 1 mM arsenate at ten temperatures. Heating caused decreased detection through protein denaturation and precipitation, modulated by lipoamide. **b**, Volcano plot of thermal stabilization *z* scores (mean, *n* = 3 experiments) and FDRs following treatment with lipoamide and arsenate. Broken vertical and solid horizontal lines show the *z* score (±1.5) and FDR (<0.05) cutoffs, respectively, classifying stabilized (green) and destabilized (blue) proteins. The positions of DLD, SFPQ, SRSF1 and several stress granule proteins are indicated. **c**, Violin and box plots showing the proportions of IDRs in each protein, categorized into stabilized (*z* > 1.5, FDR < 0.05; 70 proteins), destabilized (*z* < 1.5, FDR < 0.05; 144 proteins) and unaffected (5,811 proteins). Box plots show the median (bold bar), 25th and 75th percentiles and outliers (closed dots), and whiskers extend to the most extreme values. *P* values were determined by a two-tailed Wilcoxon signed-rank test, followed by a Holm’s test. **d**, Mean ± s.d. enrichment (>0) or depletion (<0) of each amino acid in the IDRs of stabilized (green) and destabilized (blue) proteins compared to IDRs from all 6,025 detected proteins. *P* values were determined by unpaired *t*-test, followed by a Bonferroni test. **e**, Representative G3BP1 immunofluorescence images of HeLa cells (more then three independent experiments) depleted of SFPQ or SRSF1 and treated with 10 µM lipoamide or 0.1% DMSO for 1 h, followed by 1 mM arsenate for 1 h in the presence of lipoamide. **f**, Mean ± s.d. of the percentage of stressed HeLa cells with three or more G3BP1^+^ stress granules (SGs); *n* = 292–615 cells from three independent experiments. Dots indicate the mean values from each experiment. *P* values were determined by Tukey’s test. **g**, Representative immunofluorescence images of HeLa cells (more than three independent experiments) treated with 1 mM arsenate for 1 h. Broken lines indicate the edges of the cytoplasm and nucleus of one example cell. Source data

**Extended Data Fig. 6:**
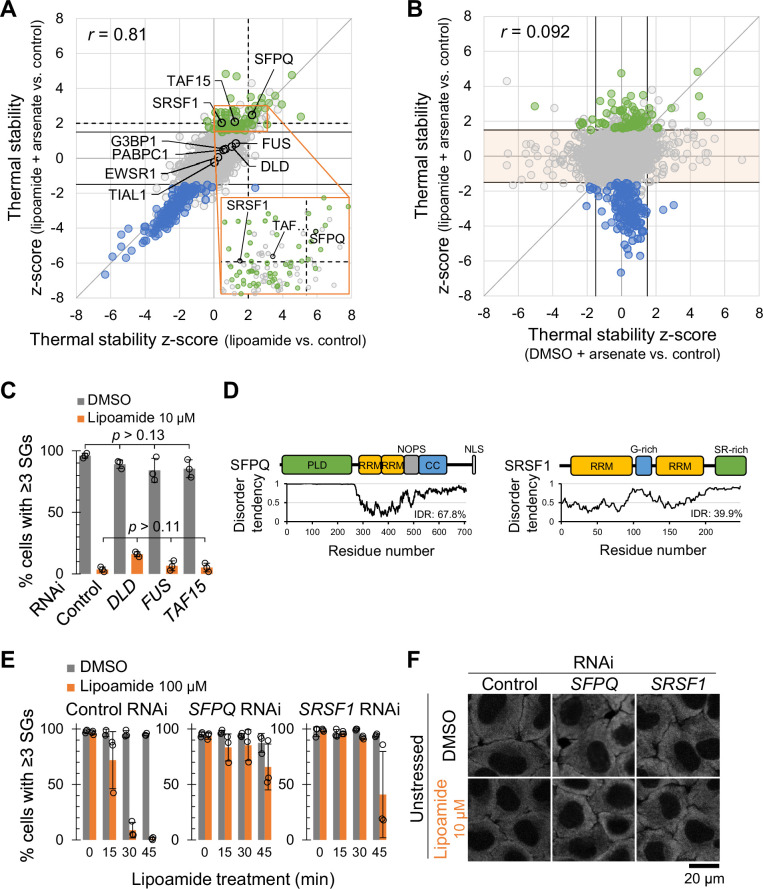
SFPQ and SRSF1 are cellular targets of lipoamide not necessary for stress granule formation. **a**, Z scores of protein thermal stability in HeLa cells treated with only lipoamide and both lipoamide and arsenate. Proteins categorized as stabilized and destabilized in Fig. 4c, d are depicted in green and blue, respectively. The positions of FUS, TAF15, DLD, SFPQ, and SRSF1 are indicated. Black solid and broken lines indicate cutoffs of z scores used for the IDR analysis in Fig. 4c (±1.5) and the targeted RNAi screen (+2), respectively. **b**, Z scores of protein thermal stability in HeLa cells treated with only arsenate and both lipoamide and arsenate. Black lines indicate |z-score| = 1.5. In most proteins with Increased or decreased thermal stability by only arsenate treatment (|z-score [arsenate]| > 1.5), the shifts were prevented by lipoamide pre-treatment (|z-score [arsenate + lipoamide]| < 1.5; masked in orange). Proteins categorized in stabilized and destabilized in Fig. 4c, d are depicted in the same colors; note that shifts in their thermal stability was not primarily due to treatment with arsenate. **c**, Mean ± s.d. of percentage of HeLa cells with ≥ 3 G3BP1-positive stress granules. Cells depleted of indicated genes were treated with 10 µM lipoamide or 0.1% DMSO for 1 h followed by 1 mM arsenate for 1 h in the presence of lipoamide before stained with G3BP1. *n* = 324–393 cells from 3 independent experiments. *p* values by Tukey’s test with no multiple comparison corrections. **d**, Domain compositions and distributions of IDRs of human SFPQ (left) and SRSF1 (right). PLD, prion-like domain; RRM, RNA recognition motif; NOPS, NonA/paraspeckle domain; CC, coiled-coil domain; NLS, nuclear localizing signal; G-rich, glycine-enriched domain; SR-rich, serine/arginine-enriched domain. **e**, Mean ± s.d. of percentage of cells with ≥3 stress granules. HeLa cells depleted of indicated genes were treated with 3 mM arsenate for 1 h, and then with 100 µM lipoamide or the control DMSO in the presence of arsenate for indicated minutes. *n* = 213–467 cells from 3 independent experiments. **f**, Representative images of HeLa cells treated and labeled as in Fig. 4e but without arsenate, representative of 3 independent experiments. Source data

As the effects of lipoamide on FUS in vitro were likely too small to explain the effect of lipoamide on stress granule dynamics in cells, we turned to cellular thermal proteome profiling (TPP). Here, aliquots of HeLa cells treated with DMSO (solvent control), lipoamide, arsenate or lipoamide and arsenate were heated to a range of different temperatures, and the abundance of soluble proteins was measured by quantitative mass spectrometry. A relative increase in abundance with temperature is indicative of protein thermal stability^32,33^ (Fig. 3a), summarized as *z* scores (Fig. 3b and Extended Data Fig. 6a,b). Increased protein thermal stability in the presence of a small molecule often indicates interaction^34,35^. Thermal stabilities of proteins in lipoamide- versus lipoamide-and-arsenate-treated cells showed a strong positive correlation, but arsenate- versus lipoamide-and-arsenate-treated cells did not, indicating a dominant effect of lipoamide. Furthermore, lipoamide treatment also broadly reversed the thermal stability changes that occurred due to arsenate treatment (Extended Data Fig. 6a,b). Therefore, we focused on analysis of the sample treated with both lipoamide and arsenate. As a positive control, we confirmed that the thermal stability of DLD was weakly but significantly increased, consistent with lipoamide binding to the active site (*z* = 0.66 ± 0.007; adjusted *P* value false discovery rate (FDR) = 2.6 × 10^−4^). As we would predict from its mitochondrial localization and enzymatic function, RNA interference (RNAi) of DLD affected neither stress granule formation nor the lipoamide activity to prevent it (Extended Data Fig. 6c).

Many proteins had higher TPP z scores than DLD (Fig. 3b). Lipoamide and arsenate treatment resulted in significantly increased thermal stability of 70 proteins and reduced the thermal stability of 144 proteins compared to no treatment (Fig. 3b). Histone deacetylase 1 (HDAC1) and HDAC2 were also stabilized (*z* = 3.58 and FDR = 1.8 × 10^−14^ and *z* = 4.75 and FDR = 7.4 × 10^–5^, respectively), consistent with a recent report^36^. Stabilized proteins had disproportionately long IDRs that contained an excessively high proportion of arginine, tyrosine and phenylalanine residues, whereas destabilized proteins showed the opposite trend (Fig. 3b–d). Arginine- and tyrosine-rich IDRs are characteristic of stress granule proteins, such as the FET family (FUS, TAF15 and EWSR1)^37^, although their individual thermal stability was not statistically significantly increased (*z* = 0.84 ± 1.3, FDR = 0.73; *z* = 2.08 ± 1.5, FDR = 0.20; *z* = 0.05 ± 0.82, FDR = 0.87, respectively). This is consistent with no strong interaction between FUS and lipoamide in vitro. Also, individual FET family proteins are not necessary for stress granule formation^10^ nor lipoamide activity (Extended Data Fig. 6c), further indicating that they are not primary targets of lipoamide for stress granule dissolution.

### Two stress granule proteins are necessary for lipoamide activity

To assess which of the proteins identified as interacting with lipoamide by TPP are necessary for lipoamide activity, we performed an endoribonuclease-prepared small interfering RNA (esiRNA)^38^-mediated gene knockdown screen of all 122 proteins with increased thermal stability (*z* > 2; Supplementary Table 1 and Extended Data Fig. 6a). We looked for genes whose depletion reduced lipoamide activity in preventing stress granule formation, which identified only two proteins: splicing factor proline- and glutamine-rich (SFPQ) and splicing factor serine/arginine-rich splicing factor 1 (SRSF1)^39,40^. Lipoamide pretreatment partially but robustly failed to prevent stress granule formation in cells with SRSF1 knockdown and almost completely failed to prevent stress granule formation in cells with SFPQ RNAi knockdown (Fig. 3e,f and Extended Data Fig. 6d). These were the only two confirmed hits; no other knockdown showed necessity of that protein for lipoamide activity. RNAi of SFPQ or SRSF1 also prevented dissolution of pre-existing stress granules following lipoamide treatment (Extended Data Fig. 6e). Stress granule formation was neither exacerbated in stressed cells nor induced in nonstressed cells by these RNAis, showing that the phenotype of lipoamide-pretreated cells is not simply due to a basal increase in stress granule formation (Fig. 3e,f and Extended Data Fig. 6f).

We also tried to identify compounds that engage with lipoamide by using the click-cross-link analog (Fig. 1c) using UV-induced cross-linking to nearby proteins in cells and conjugating a streptavidin tag using click chemistry to affinity purify and identify these proteins. However, as we failed to detect the expected positive-control DLD, we instead focused on the results of TPP.

Similar to FUS and TDP-43, SFPQ and SRSF1 are nuclear-localizing proteins and, in stressed cells, also localize to stress granules (Fig. 3g). Therefore, lipoamide-dependent stress granule dissolution is dependent on two IDR-containing stress granule proteins.

### SFPQ redox state mediates stress granule dissolution

**Fig. 4:**
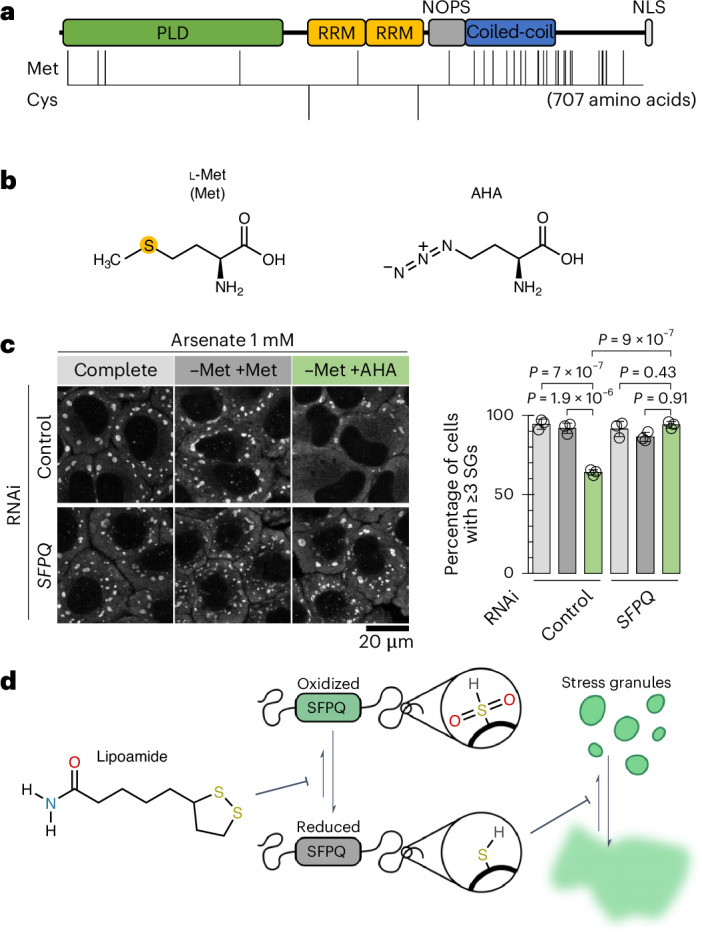
SFPQ redox state may mediate lipoamide activity. **a**, Schema of the distributions of methionine (Met; 28 residues) and cysteine (Cys; 2) residues in human SFPQ; PLD, prion-like domain; RRM, RNA recognition motif; NOPS, NonA/paraspeckle domain; NLS, nuclear localization signal. **b**, Chemical structures of l-methionine and its nonnatural analog AHA. **c**, Left, representative images of HeLa cells subjected to the indicated RNAi knockdowns and cultured in complete medium (light gray) or methionine-free medium supplemented with 1 mM methionine (dark gray) or AHA (green) for 2 h, followed by 1 mM arsenate for 1 h (the experimental schematic is presented in Extended Data Fig. 7e). Stress granules were labeled with G3BP1. Right, mean ± s.d. of the percentage of stressed HeLa cells with three or more G3BP1^+^ SGs; *n* = 325–407 cells from three independent experiments. *P* values were calculated by a Tukey test without multiple-comparison correction. **d**, Schema of SFPQ as a redox sensor to modulate stress granule condensation. Source data

Lipoamide activity requires the redox-active dithiolane (Fig. 2j), and SFPQ is notably rich in redox-sensitive methionine (28 of 707 amino acids; Fig. 4a). SRSF1 is not methionine rich, with only 3 of 248 amino acids. Pioneering work has previously shown that methionine oxidation modulates the function and material properties of phase-separated yeast ataxin-2 (ref. ^41^), and methionine oxidation in SFPQ has been detected in cells^42^. These findings suggest that SFPQ may be a main target of lipoamide in a redox-based mechanism of action.

**Extended Data Fig. 7:**
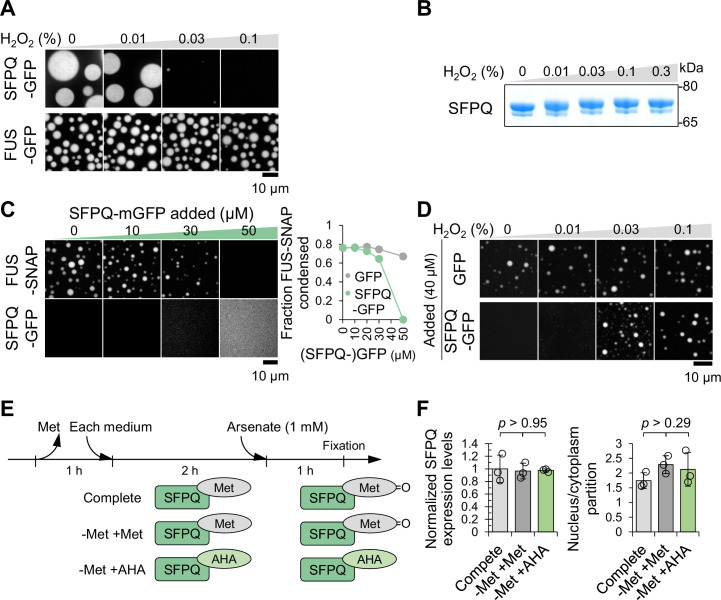
SFPQ dissolves FUS condensates *in vitro.* **a**, Representative images of SFPQ-GFP (top, 10 µM) and FUS-GFP (bottom, 7 µM) protein condensates at a low salt condition (75 mM KCl) and in the presence of H_2_O_2_ from >3 independent experiments. **b**, SDS-PAGE (non-reduced condition) of 10 µM of the purified and untagged SFPQ proteins in diluted state oxidized with the indicated percentages of H_2_O_2_ for 30 min. **c**, Left, representative images of co-incubation of indicated concentrations of SFPQ-GFP and 6 µM of FUS-SNAP at a physiological salt concentration (150 mM KCl) from >3 independent experiments. SFPQ-GFP do not form condensates at 150 mM KCl while they suppress condensation of FUS proteins in dosage-dependent manner. Right, mean ± s.d. of FUS condensate fraction in the presence of GFP (control) or SFPQ-GFP. *n* = 16 image fields. **d**, Representative images of FUS-SNAP condensates (4 µM) co-incubated with 40 µM of GFP or SFPQ-GFP at a physiological salt concentration (150 mM KCl) in the presence of indicated percentages of H_2_O_2_ from 3 independent experiments. **e**, Schema of the time course used in Fig. 5c. Cells were firstly cultured in methionine (Met)-free medium and then in each medium (complete medium or Met-free medium supplemented with Met or AHA) before arsenate treatment. **f**, Left, mean ± s.d. of relative expression levels of SFPQ normalized to those of α-Tubulin in HeLa cells, detected by immunoblotting from 3 independent experiments. Right, mean ± s.d. of nucleus/cytoplasm signal ratio of SFPQ in HeLa cells, detected by immunostaining from 3 independent experiments (*n* = 363–402 cells). The cells were treated with the indicated medium as in E. p values from Tukey’s test. Source data

We used in vitro experiments with purified SFPQ to analyze the effect of oxidation on SFPQ on condensate formation by using the oxidizing agent hydrogen peroxide (H_2_O_2_). SFPQ condensation was induced at low-salt concentrations (75 mM KCl; Extended Data Fig. 7a). Oxidation of SFPQ, confirmed by modulated electrophoretic migratory aptitude in nonreducing SDS–PAGE (Extended Data Fig. 7b), led to dissolution of SFPQ condensates in an H_2_O_2_-concentration-dependent manner (Extended Data Fig. 7a), similar to the behavior of ataxin-2 condensates^41^. This showed that oxidation alters the phase separation properties of SFPQ–GFP proteins. By contrast, H_2_O_2_ alone did not lead to GFP-tagged FUS (FUS–GFP) condensate dissolution (Extended Data Fig. 7a). Therefore, oxidation-mediated condensate dissolution is specific to a subset of proteins.

We next asked if SFPQ and its oxidation state could affect FUS–SNAP condensates and tested FUS condensation at 150 mM KCl, which kept SFPQ–GFP proteins in an uncondensed state (Extended Data Fig. 7c). We found that adding SFPQ–GFP prevented FUS–SNAP condensation (Extended Data Fig. 7c). This effect did not occur by adding only GFP protein (Extended Data Fig. 7c). H_2_O_2_ treatment allowed FUS condensate formation in the presence of SFPQ (Extended Data Fig. 7d). This minimal in vitro model indicates that SFPQ proteins dissolve stress granule protein condensates in a redox-state-dependent manner.

Based on these in vitro results, we hypothesized that stress granule formation would be attenuated if SPFQ is not oxidizable. To assess this, we need mutant cells expressing SFPQ where methionine is replaced with another hydrophobic amino acid. However, SFPQ appears to be vital, as we were unable to generate an SFPQ deletion line, and we were unable to establish mutant SFPQ-expressing cell lines depleted of endogenous SFPQ. Therefore, we instead aimed to replace methionine with a nonoxidizable, nonnatural analog l-azidohomoalanine (AHA), which is normally used for protein labeling^43^ (Fig. 4b). Cells were cultured in methionine-free medium supplemented with AHA for 2 h, resulting in methionine-to-AHA replacement in newly synthesized proteins, and then stressed with arsenate for 1 h (Extended Data Fig. 7e). AHA treatment affected neither SFPQ protein levels nor its nuclear–cytoplasmic distribution but resulted in attenuated stress granule formation (Fig. 4c and Extended Data Fig. 7f). As incorporation of AHA is not specific to SFPQ, we tested whether the effect on stress granule formation was SFPQ dependent. Indeed, normal stress granule formation was rescued by depletion of SFPQ (Fig. 4c). This suggests that SFPQ in the reduced state is responsible for preventing stress granule formation in the presence of lipoamide. One possibility is that cell stress leads to oxidation of SFPQ, allowing stress granule condensation. In this scenario, lipoamide reduces SFPQ and restores its stress granule dissolution activity (Fig. 4d).

### Lipoamide rescues nuclear FUS and TDP-43 functions

**Fig. 5:**
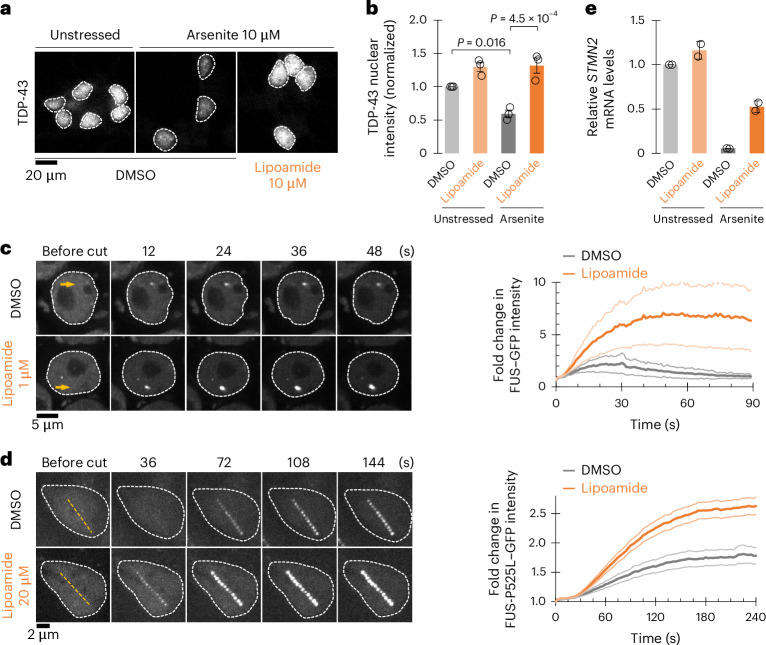
Lipoamide improves nuclear localization of FUS and TDP-43. **a**, Representative images of iPS cell-derived MNs from three independent experiments treated with 0.1% DMSO or 10 µM lipoamide for 1 day, followed by 10 µM arsenite for 5 days in the presence or absence of lipoamide and labeled with TDP-43. The broken lines indicate the outlines of the nuclei. **b**, Mean ± s.e.m. of nuclear TDP-43 levels normalized to those of unstressed DMSO-treated MNs (control); *n* = 417–1,741 cells from three independent experiments. *P* values were determined by a Tukey test. **c**, Left, images showing recruitment of FUS–GFP to sites of UV laser-induced DNA damage (yellow arrow) in the nuclei (outlined with broken lines) of iPS cells at the indicated times after laser irradiation. Cells were analyzed after 1 h of treatment with lipoamide, followed by 1 h of arsenate stress. Right, mean ± s.d. of relative FUS–GFP signal intensity in response to DNA damage; *n* = 5 (DMSO) and 7 (lipoamide) cells. **d**, Left, images of nuclei (outlined with broken lines) of iPS cell-derived MNs expressing FUS-P525L–GFP from three independent experiments cultured for 21 days and then treated with 0.02% DMSO or 20 µM lipoamide for 24 h at the indicated times after laser irradiation. Yellow lines indicate laser-irradiated sites. Right, mean ± s.e.m. of the relative intensity of FUS–GFP at sites of DNA damage after ablation; *n* = 14 (DMSO) and 18 (lipoamide) cells from three independent experiments. **e**, Mean ± s.d. of relative full-length *STMN2* mRNA levels normalized to those of *GAPDH* from two independent experiments. In **b** and **e**, MNs were treated as in **a**. Source data

**Extended Data Fig. 8:**
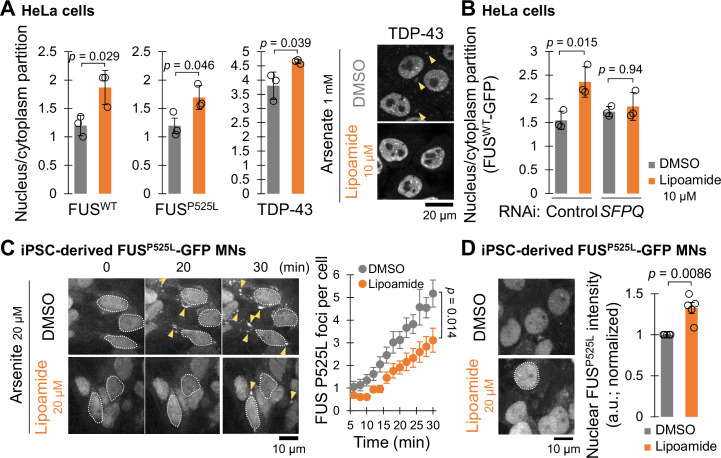
Lipoamide rescues nuclear localization and functioning of ALS-linked proteins. **a**, Left, mean ± s.d. of nuclear-cytoplasmic intensity ratio of FUS and TDP-43 in HeLa cells pre-treated with 10 µM lipoamide (0.1% DMSO as the control) for 1 h followed by 1 mM arsenate for 1 h in the presence of lipoamide. *n* = 290–603 cells from 3 independent experiments. *p* values by two-tailed unpaired *t*-test. Right, representative image of the HeLa cells stained with TDP-43. Arrowheads indicate some of cytoplasmic punctate signals (stress granules). **b**, Mean ± s.d. of nuclear-cytoplasmic intensity ratio of FUS-GFP in SFPQ-depleted and the control HeLa cells pre-treated with 10 µM lipoamide (0.1% DMSO as the control) for 1 h followed by 1 mM arsenate for 1 h in the presence of lipoamide. *n* = 450–543 cells from 3 independent experiments. *p* values by Tukey’s test. **c**, (Left) time-lapse images of iPSC-derived MNs expressing FUS P525L-GFP cultured for 14 days. Cells were treated with 0.02% DMSO or 20 µM lipoamide for 1 h followed by 20 µM arsenite for indicated minutes in the presence of lipoamide. Broken lines indicate outline of some nuclei. Arrowheads indicate some cytoplasmic FUS P525L foci. (Right) mean ± s.e.m. of number of FUS P525L foci per MN after arsenite treatment. *n* = 16 (DMSO) and 18 (lipoamide) cells from 3 independent experiments. *p* value by two-tailed unpaired *t*-test. **d**, (Left) representative images of iPSC-derived MNs expressing FUS P525L-GFP cultured for 5 days and then 30 days in the presence of 0.02% DMSO or 20 µM lipoamide. Broken lines indicate outline of some nuclei. (Right) mean ± s.e.m. of nuclear intensity of FUS P525L-GFP, normalized to that in the control (DMSO). *n* = 64–198 cells from 5 independent experiments. *p* value by one-sample unpaired *t*-test. Source data

FUS and TDP-43 have important nuclear functions in unstressed cells. We asked whether lipoamide treatment not only dissolves stress granules but also returns these proteins to the nucleus. Similar to FUS–GFP (Fig. 2a), lipoamide pretreatment increased partitioning of TDP-43 and the ALS-associated nuclear localization sequence (NLS) mutant FUS-P525L–GFP to the nucleus in stressed HeLa cells (Extended Data Fig. 8a). Such partitioning of FUS back to the nucleus was suppressed in SFPQ-depleted cells, suggesting that prevention of stress granule formation facilitates nuclear relocalization of FUS proteins (Extended Data Fig. 8b). To confirm that the effects on nuclear accumulation also occur in cells prominently defective in ALS, we analyzed iPS cell-derived MNs. Lipoamide had a similar effect on nuclear partitioning of WT TDP-43 in stressed (prolonged oxidative stress with a low dose (10 µM) of arsenite) and FUS-P525L–GFP in nonstressed but long-term culture conditions (Fig. 5a,b and Extended Data Fig. 8c,d)^44^.

We characterized the functional importance of relocalization to the nucleus by considering FUS and TDP-43 nuclear functions. FUS forms condensates at sites of DNA damage to engage in DNA damage repair, and this malfunction caused by ALS-linked mutations in FUS is thought to underlie neuronal dysfunction in ALS^45^. Lipoamide increased recruitment of FUS–GFP (WT in iPS cells and P525L mutant in iPS cell-derived MNs) to laser-induced DNA damage foci (Fig. 5c,d). TDP-43 contributes to normal transcript splicing in the nucleus, particularly of stathmin-2 (*STMN2*) transcripts, and altered *STMN2* splicing leading to reduced transcript levels is a hallmark of ALS^6,46^. In iPS cell-derived MNs, prolonged oxidative stress recapitulated reduced *STMN2* mRNA levels. This reduction was not fully, but robustly, rescued by lipoamide treatment, concomitant with TDP-43 nuclear partitioning (Fig. 5b,e). Lipoamide action therefore dissolves stress granules, allows return of ALS-linked proteins to the nucleus and restores nuclear functions of FUS and TDP-43.

### Lipoamide alleviates ALS phenotypes in familial ALS models

**Fig. 6:**
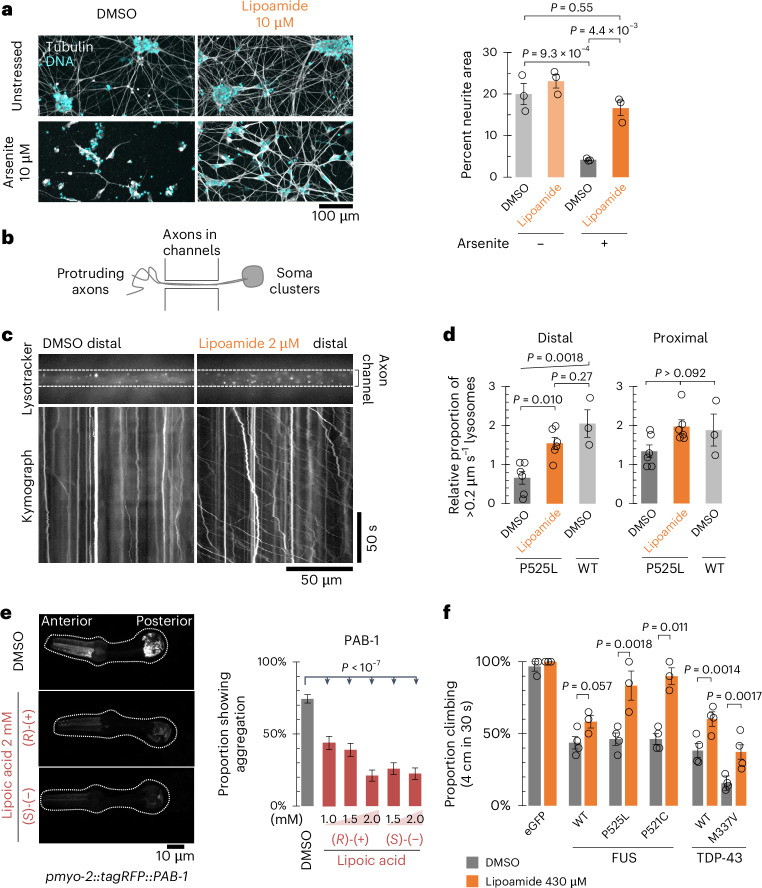
Lipoamide improves cellular fitness in ALS iPS cell-derived MN and animal disease models. **a**, Left, representative images of iPS cell-derived MNs treated as in Fig. 5a. Right, mean ± s.e.m. of the percentage of neurite (tubulin positive) area. Eighteen image fields were acquired from three independent experiments. *P* values were determined by a Tukey test without multiple-comparison correction. **b**, Schematic of a neuron culture showing the channels through which axons grow from the soma on the right. **c**, Kymographs of lysosome movement in the distal portion of FUS-P525L MN axons 3 days after treatment with compound solvent (DMSO) or 2 μM lipoamide, visualized with lysotracker. **d**, Mean ± s.e.m. of the relative proportion of lysotracker-labeled lysosomes moving at an average speed of greater than 0.2 μm s^–1^ following 3 days of treatment with 2 μM lipoamide or equivalent DMSO concentration solvent control for iPS cell-derived MNs expressing either FUS-P525L or WT FUS, normalized to the mean of proportion moving (proximal and distal) in DMSO-treated FUS-P525L MNs; *n* = 6 (FUS-P525L) or 3 (WT) biological replicates, analyzing five axon bundles per replicate. *P* values were determined by a Tukey test without multiple-comparison correction. **e**, Left, representative images of the pharynx of worms expressing fluorescently tagged PAB-1 with or without lipoic acid treatment (2 mM). Broken lines indicate the edges of the pharynges. Right, mean ± s.e.m. of the incidence of each protein aggregation in the pharyngeal muscles. Incidence of PAB-1 aggregation was scored from the proportion of animals with more than ten aggregates. *P* values were determined by two-tailed Fisher’s exact test; *n* = 107 to 230 for each sample from *n* = 1 experiment. **f**, Mean ± s.e.m. of the proportion of flies that climbed that were fed 0.1% DMSO (solvent control) or 430 µM lipoamide. Human WT or ALS-linked mutants of FUS (left) or TDP-43 (right) were expressed in MNs. *P* values were determined by unpaired two-tailed *t*-test; *n* = 30–40 (FUS) and 130–202 (TDP-43) flies from *n* = 3 or 4 independent experiments. Source data

The ultimately lethal phenotype of ALS is thought to be caused by axon defects in MNs. Indeed, iPS cell-derived MNs expressing FUS-P525L show a MN survival defect in vitro, with reduced neurite growth and defective axonal transport^44^. Lipoamide treatment rescued neurite growth of iPS cell-derived MNs stressed with arsenite, as shown by increased area covered in neurites in a nonpolarized culture (Fig. 6a). We tested if this correlated with improved axonal transport by tracking lysosome transport in iPS cell-derived MN axons grown in silicone channels (Fig. 6b). As previously observed, under nonstressed conditions, distal axonal transport of lysosomes was reduced by the expression of FUS-P525L^44^, and lipoamide recovered transport to a similar level as that observed in WT FUS iPS cell-derived MNs (Fig. 6c,d). MN degeneration caused by an ALS-associated FUS mutant can therefore be rescued by lipoamide.

**Extended Data Fig. 9:**
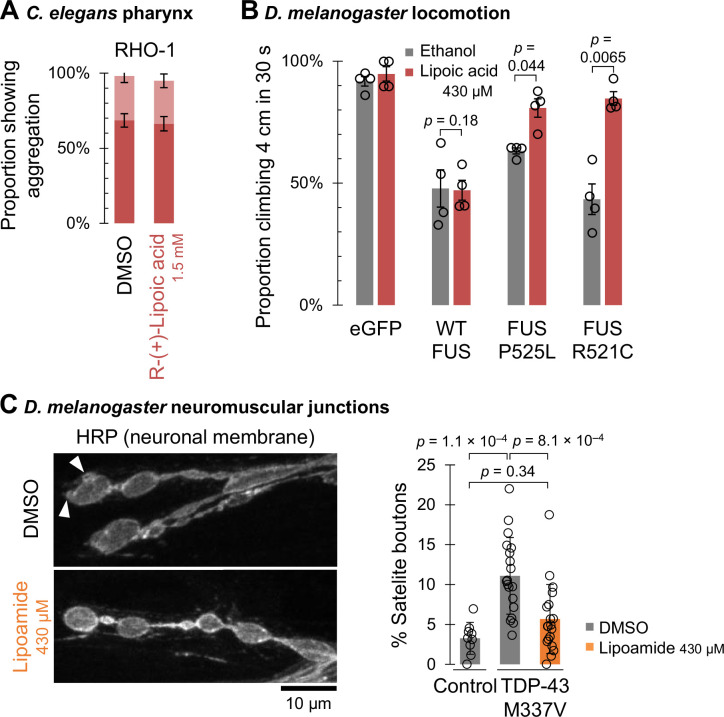
Extended analysis of *C. elegans* and *D. melanogaster* animal models of ALS. **a**, Mean ± s.e.m. of incidence of each protein aggregation in the pharyngeal muscles. Incidence of RHO-1 was scored on a low, medium, high scale (see Methods). **b**, Mean ± s.e.m. of percentage of flies that climbed, with lipoic acid feeding in place of lipoamide in Fig. 6f. *p* values by unpaired two-tailed *t*-test. **c**, (Left) Representative images of synaptic boutons of TDP-43 M337V-expressing flies, immunostained with an antibody against horseradish peroxidase (HRP), which labels the neuronal membrane. Arrowheads indicate appearance of satellite boutons. (Right) mean ± s.d. of percentage of satellite boutons (number of satellite boutons/number of total boutons) per fly. The control flies fed with 0.1% DMSO (gray; *n* = 9) and TDP-43 M337V-expressing flies fed with 0.1% DMSO (*n* = 19) or that containing 430 µM lipoamide (orange; *n* = 19) were examined. *p* value by Tukey’s test. Source data

Aggregation of TDP-43 and FUS in neurons is a hallmark pathology of ALS, and aggregation of proteins is also a phenotype of aging more generally, including in *Caenorhabditis elegans*^47^. PAB-1, the *C. elegans* ortholog of the human stress granule protein PABPC1, forms reversible stress granules following heat shock and accumulates into large solid aggregates following mild chronic temperature stress or during aging^47,48^. Feeding lipoic acid (selected as it had higher solubility in food medium than lipoamide) caused a dose-dependent reduction in the number of aggregates of transgenic PAB-1 (Fig. 6e), but not those of a non-stress granule protein RHO-1 (Extended Data Fig. 9a)

In *Drosophila melanogaster*, MN-specific expression of human FUS and TDP-43 induces ALS-like phenotypes, including motor defects manifesting as a reduced ability for negative geotaxis^49,50^. Feeding either lipoamide or lipoic acid improved climbing ability in flies expressing FUS NLS mutants, either FUS-P525L or FUS-R521C (Fig. 6f and Extended Data Fig. 9b). Similarly, lipoamide feeding alleviated climbing defects in flies expressing TDP-43, either WT or the ALS-linked mutant TDP-43-M337V (Fig. 6f). The severe phenotype caused by TDP-43-M337V was associated with abnormal neuromuscular junction morphology and the presence of satellite boutons, similar to previously described phenotypes of another ALS-linked mutant TDP-43-G298S^51^. Lipoamide treatment suppressed the appearance of satellite boutons (Extended Data Fig. 9c). Collectively, our data show that lipoamide can alleviate ALS-like phenotypes in human-derived MNs and animal models caused by the expression of two ALS-associated stress granule protein mutants.

## Discussion

Stress granules are an example of a liquid cellular compartment formed by phase separation. Due to the strong genetic association between ALS and stress granule proteins, we sought to identify small molecules that alter the physiological function of stress granule proteins in forming biological condensates. Our screen identified lipoamide, which partitioned into stress granules in cells and FUS protein condensates, as an in vitro model of stress granules. Lipoamide caused rapid disassembly of existing stress granules and prevented the formation of new stress granules. Our SAR data showed that the potency of lipoamide for stress granule dissolution could be increased, particularly by monosubstituted amines and some alkane backbone modifications, perhaps subtly modulating ditholane redox potential and kinetics, although not to the nanomolar level often sought in drug candidate development.

The dithiolane is always required for activity of lipoamide-like molecules. However, the degree to which other areas of lipoamide could be modified while retaining activity was large. This shows that the lipoamide compound family has the potential for medicinal chemistry development. The high degree to which the nondithiolane regions could be altered would be surprising for a molecule that binds a protein at a structured binding site, although this is perhaps unsurprising given that TPP indicated the interaction of lipoamide with intrinsically disordered proteins. Overall, lipoamide is therefore likely acting by delivering a redox-active dithiolane payload to physicochemical environments formed by its interacting proteins, including stress granules. More active lipoamide derivatives may be improving targeting (cell uptake and partition into stress granules) while leaving the dithiolane payload intact.

Discovery of lipoamide has allowed us to identify a key protein likely sensing the redox state of the cell. Among all the proteins stabilized by lipoamide, our RNAi screen showed that only SFPQ and SRSF1 were necessary for the rapid (<20 min) lipoamide effect on stress granule dynamics. Both SFPQ and SRSF1 are stress granule proteins. SFPQ is very methionine rich, which likely confers high redox sensitivity not found in most arginine/tyrosine-rich IDR-containing proteins. SFPQ activity was indeed methionine and redox dependent. SFPQ appears to be the primary target for early lipoamide activity, which overrides the ability of SFPQ to act as an oxidation sensor, promoting stress granule dissolution only when reduced. Although we saw some effect of lipoamide on FUS condensate liquidity in vitro, we suspect that this is a similar effect of secondary importance on a less redox-sensitive stress granule protein or a minor effect from strong partitioning of lipoamide into this condensate.

SFPQ is an ALS-associated protein. SFPQ is often observed to be mislocalized from the nucleus to the cytoplasm in disease model neurons, and two missense mutations (N533H and L534I) in SFPQ were found in individuals with familial ALS, which affected axonal morphology and SFPQ localization^52–55^. It is possible that altered SFPQ dynamics and activity could modulate cellular responses to redox state in ALS tissues. Overall, our identification of lipoamide allowed us to dissect a mechanism where dissolution of stress granule condensates involves direct redox sensitivity of a key stress granule protein. Lipoamide therefore represents productive small-molecule intervention in an emerging paradigm: redox sensitivity of proteins able to phase separate as a homeostatic mechanism^41,56,57^.

Oxidative stress is a common theme in ALS pathogenesis mechanisms^58–60^. We showed that lipoamide can recover pathology in MNs and animals expressing ALS-associated stress granule mutants (FUS and TDP-43) with no explicit oxidative stress. This leaves the link between the redox-associated cellular effects of lipoamide on stress granules and neuron/animal model outcomes ambiguous but consistent with modulated stress granule formation in response to stochastic oxidative stresses. Previous works described that HDAC1 and HDAC2 are responsible for long-term changes in histone acetylation in MNs^61^ and that they are inhibited by lipoamide^36^. However, we did not find them necessary for short-term lipoamide activity on stress granules, instead detecting the two stress granule proteins both necessary for lipoamide activity. However, this does not preclude synergistic long-term nuclear effects. FUS NLS mutations are strongly associated with ALS^44,62–64^, and dissolution of stress granules by lipoamide leads to the return of FUS and TDP-43 to the nucleus. Indeed, we saw that lipoamide does not prevent nuclear FUS condensation and rescues nuclear TDP-43 functions. Therefore, we suggest that lipoamide-dependent dissolution of stress granules is beneficial for proper nuclear localization of ALS-linked proteins and thus nuclear function, leading to suppressed ALS-linked phenotypes.

Although lipoamide does not possess the characteristics of a typical therapeutic, it is notable that lipoic acid is used to treat diabetic neuropathy, and, in humans, a 600-mg daily dose yields plasma concentrations of 8 to 30 μM (refs. ^65,66^). This is comparable to the concentrations used in our cell-based assays, and we saw beneficial effects in human-derived MNs and *D. melanogaster* models of ALS. Therefore lipoamide, in addition to allowing for the discovery of direct redox sensation by SFPQ for stress granule dissolution, has some plausibility as the basis of a therapeutic with medicinal chemistry potential for further improvements. However it is important to point out that we have not shown a direct relationship between stress granule dissolution and phenotype rescue in our disease models. Future work will be required to understand the relationship between stress granule formation and disease in animal models; the identification of lipoamide provides a powerful tool to begin such investigations.

## Methods

### Cells and cell lines

Kyoto HeLa cells were maintained in high-glucose DMEM (Thermo Fisher Scientific) supplemented with 10% fetal bovine serum (FBS) and 1% penicillin–streptomycin at 37 °C with 5% CO_2_. Stable Kyoto HeLa BAC cell lines expressing proteins with a C-terminal fluorescent protein were generated using BAC recombineering^67^. This yields near-endogenous expression levels of the fusion protein^12,68^. In these lines, GFP is part of a modified localization and affinity purification (LAP) tag^69^, providing a short linker. Stable expression was kept under G418 selection (400 μg ml^–1^; Gibco). The following BAC lines were used: FUS (MCB_005340; also used for the compound screen), COIL (MCB_0002582), DCP1A (MCB_0003876), EWSR1 (MCB_0008863), PABPC1 (MCB_0006901), TIAL1 (MCB_0008967) and TRP53BP1 (MCB_0003740). HeLa FUS-P525L–GFP cells were generated using the same approach as described previously for the iPS cell lines^44^.

Human iPS cells were grown in either TeSR E8 or mTeSR1 medium (Stem Cell Technologies) at 37 °C with 5% CO_2_ (ref. ^70^). iPS cells lines, derived from two different donors, expressing FUS with a C-terminal GFP fluorescent marker were used. All were generated using CRISPR–Cas9-assisted tagging and mutagenesis and have been previously described^44^. KOLF iPS cell lines expressing WT FUS–GFP or FUS-P525L–GFP were previously generated from the KOLF-C1 clonal iPS cell line produced as part of the Human Induced Pluripotent Stem Cell Initiative^71^. KOLF-C1 cells were derived from a healthy male donor. In these lines, GFP is part of a modified LAP tag^69^, yielding an identical fusion protein sequence to the Kyoto HeLa BAC cell line. AH-ALS1-F58 iPS cells expressing FUS-P525L with a C-terminal GFP fluorescent marker were previously generated from a clonal iPS cell line from a female individual with ALS expressing FUS-P521C. The P525L mutation and GFP tag were introduced and the P521C mutation was corrected by simultaneous tagging and mutagenesis^44,72,73^.

MNs used for the study of FUS-P525L dynamics were induced and maintained as described previously^74^. MNs used for the prolonged arsenite stress assay were derived from commercially available WTC-11 iPS cells (Coriell Institute, GM25256) and differentiated as described previously^75^. MNs used for axonal lysosome mobility assays were generated from AH-ALS1-F58 iPS cells expressing FUS-P525L. In short, the iPS cells were differentiated into neuronal progenitor cells and matured to spinal MNs in Matrigel-coated plates, as previously described^44,70^. The coating and assembly of silicone microfluidic chambers (MFCs; RD900, Xona Microfluidics) to prepare for subsequent seeding of MNs was performed as described previously^44,76,77^. MNs were eventually seeded into one side of an MFC for maturation to obtain a fully compartmentalized culture with proximally clustered somata, and their dendrites were physically separated from their distal axons, as only the latter type of neurite was able to grow from the proximal seeding site through a microgroove barrier of 900-µm-long microchannels to the distal site (Fig. 6b). All subsequent imaging in MFCs was performed at day 21 of axonal growth and MN maturation (day 0 was the day of seeding into MFCs).

All procedures using human cell samples were performed in accordance with the Helsinki convention and approved by the Ethical Committee of the Technische Universität Dresden (EK45022009, EK393122012).

### Recombinant protein purification

Recombinant proteins were purified using baculovirus/an insect cell expression system, as previously described^12^. Briefly, 6×His-MBP-FUS–GFP and 6×His-MBP-FUS–SNAP were purified from Sf9 cell lysates by Ni-NTA (Qiagen) affinity purification. The 6×His-MBP tag was cleaved by 3C protease, concentrated by dialysis and further purified by size-exclusion chromatography. 6×His-MBP-SFPQ–GFP was purified from Sf9 cell lysates by affinity purification using Ni-NTA and amylose resin (New England Biolabs). The 6×His-MBP tag and, if necessary, the GFP tag were cleaved by 3C protease and TEV protease, respectively, and the target proteins were concentrated by dialysis and further purified by cation exchange chromatography. The composition of the storage buffer for the purified proteins was 1 M or 500 mM KCl, 50 mM Tris-HCl (pH 7.4), 5% glycerol and 1 mM DTT, and the concentration of FUS was adjusted to 30 μM in storage buffer before use.

### Small-molecule screen

For the small-molecule screen, we used the Pharmakon 1600 library of small molecules (MicroSource Discovery Systems) prepared as 10 mM solutions in DMSO. The Kyoto HeLa BAC cell line stably expressing FUS–GFP was seeded at 4,000 cells per well in 384-well plates 24 h before the assay. The cells were pretreated with 10 μM compound for 1 h and stressed with 1 mM potassium arsenate (A6631, Sigma-Aldrich). After 1 h, cells were fixed in 4% formaldehyde and stained with 1 µg ml^–1^ Hoechst 33342 and CellMask blue (1:10,000; H32720, Thermo Fisher Scientific) before being imaged on a CellVoyager CV7000 automated spinning disc confocal microscope (Yokogawa) with a ×40/1.1-NA air objective to assess FUS–GFP localization.

FUS–GFP signal was analyzed using CellProfiler^78^, and the data were processed with KNIME. The cytoplasm and nuclei were distinguished with weak (CellMask blue) and strong (Hoechst 33342) blue fluorescent signals, respectively. Particle number and sum area, granularity (at 9, 10 and 11 pixels in the cytoplasm or 1, 5, 6, 7, 8 and 9 pixels in the nucleus) scale, texture (at 10-pixel scale) and integrated signal intensity of FUS–GFP in the nucleus and cytoplasm were measured. *Z* scores (*z* = (*x* – *μ*) / *σ*, where *x* is the observed value, *μ* is the control mean, and *σ* is the control standard deviation) relative to the DMSO-treated control wells on each plate were calculated for these parameters and combined into the Mahalanobis distance. Compounds of interest were selected on the criteria of (1) treatment returned the cells to the unstressed state (that is, reduced stress granule number and increased nuclear signal), (2) a clear monotonic dose-dependent response and (3) manual prioritization by known mechanism (for example, emetine and cardiac glycosides) or implausibility as a cell-compatible compound (for example, surfactants used as topical antiseptics).

The follow-up in vitro assay of compounds on FUS–GFP condensates was assessed in a 384-well plate format. The compound volumes (in DMSO) necessary for 1, 3, 10, 30 or 100 μM final concentration were added by acoustic dispensing (Labcyte Echo 550) to 96-well plate wells containing FUS–GFP in 3 µl of 50 mM Tris-HCl (pH 7.4), 1 mM DTT and 170 mM KCl. The final DMSO concentration was 0.01 to 1%. Using a Freedom Evo 200 liquid handling workstation (TECAN), the FUS–GFP/compound mixture was diluted in 7 μl of 50 mM Tris-HCl (pH 7.4) to reach the final composition of 50 mM Tris-HCl (pH 7.4), 1 mM DTT, 50 mM KCl, indicated concentrations of each compound and DMSO and 0.7 μM FUS–GFP. Compound/FUS–GFP and assay buffer were mixed using a standardized pipetting procedure, split into four wells in clear-bottom 384-well plates and immediately imaged using a CellVoyager CV7000 automated spinning disc confocal microscope (as described above). Condensates in suspension for six fields of view were imaged as a maximum intensity projection of six focal planes at 2-μm steps per sample. Condensate number and FUS–GFP partitioned into condensates were analyzed with a fixed intensity threshold using Fiji. The number of condensates and partitioning were weakly time dependent due to condensate sedimentation and therefore normalized assuming a linear change over time by reference to DMSO controls at the start and end of each plate row.

### Compound characterization in cells

Compound effects were assessed under a variety of conditions in HeLa cells, iPS cells or iPS cell-derived MNs. Unless otherwise indicated, cells were pretreated for 1 h using 10 μM compound from 10 mM stock in DMSO (or an equal volume of DMSO control) and stressed for 1 h with 1 mM potassium arsenate in the presence of the compounds. Live cells were imaged by widefield epifluorescence using an inverted Olympus IX71 microscope with a ×100/1.4-NA Plan Apo oil immersion objective (Olympus) and a CoolSNAP HQ CCD camera (Photometrics) using a DeltaVision climate control unit (37 °C, 5% CO_2_; Applied Precision).

Various cellular stresses were achieved by replacing 1 h of potassium arsenate treatment with other conditions: 0.4 M sorbitol (S1876, Sigma-Aldrich) from a 4 M stock in water for 1 h (osmotic stress), 42 °C in normal growth medium for 30 min (heat stress) or 100 mM 6-deoxyglucose (D9761, Sigma-Aldrich) from a 1 M stock in water in glucose-free DMEM (11966025, Thermo Fisher Scientific) supplemented with 10% fetal calf serum for 1 h (glycolysis stress). Sodium arsenite (S7400, Sigma-Aldrich) was used from a 10 mM stock in water.

Other antioxidants were used by replacing lipoamide treatment: l-ascorbic acid (A4544, Sigma-Aldrich) from a 1 M stock in water, *N*-acetyl l-cysteine (017–05131, Wako) from a 100 mM stock in water, citric acid (036-05522, Wako) from a 1 M stock in water ±-α-tocopherol (209-01791, Wako) from a 100 mM stock in ethanol and taurine (201-00112, Wako) from a 100 mM stock in water.

### Compound dose responses

To assess dose-dependent effects of lipoamide on HeLa and iPS cells expressing FUS–GFP, cells were pretreated with lipoamide for 1 h followed by 1 h of treatment with 1 mM potassium arsenate, similar to the ex vivo HeLa cell screen except serial compound dilutions in medium were prepared manually from 80 μM to ~0.4 nM at 1.189× dilution steps. Small dilution steps rather than concentration replicates were selected as this approach provides greater statistical power from a set number of samples^79^. The final DMSO concentration was 0.08% in all samples, and each plate included at least 12 control wells with 0.08% DMSO. Cytoplasmic FUS–GFP condensate number and nuclear/cytoplasm partitioning of FUS–GFP were analyzed using custom macros in Fiji. Nuclei were identified by intensity thresholding of DNA images labeled with Hoechst following a 5-pixel Gaussian blur. Cytoplasmic FUS–GFP condensates were identified by intensity thresholding of FUS–GFP images following a 10-pixel weight 0.9 unsharp filter masked by the thresholded nuclei. The ratio of the number of cytoplasmic FUS–GFP condensates to that of nuclei was taken as cytoplasmic FUS–GFP condensates per cell per field of view, and *p*, the ratio of partitioning of FUS–GFP to the nucleus and the cytoplasm, was derived from *a* = *v*_*n*_ / *v*_*t*_, the ratio of nuclear to total green signal per field of view, where *p* = *a* / (1 – *a*). These data were log transformed and fitted to a Rodbard sigmoidal curve^80^ to determine EC_50_. Six fields of view were captured and analyzed per condition.

The series of lipoamide analogs including lipoamide and lipoic acid were newly synthesized by Wuxi AppTec and provided through Dewpoint Therapeutics. To assess the dose–response effects, HeLa BAC cells expressing FUS–GFP were seeded in 384-well plates (4,000 cell per well) 24 h before treatment, pretreated with the compounds in a half-log dilution series (from 30 µM to 3 nM: seven concentrations) using an Echo 650 and treated for 1 h with 1.5 mM potassium arsenate before fixation with 4% formaldehyde for 15 min, permeabilized with 0.1% Triton X-100 for 10 min and counterstained with Hoechst and CellMask blue as described above. Imaging was performed using an Opera Phenix (PerkinElmer; ×20, nine fields of view, binning 2) and Harmony 4.9 software to determine cytoplasmic FUS–GFP condensate number and cytoplasmic and nuclear FUS–GFP intensities to calculate nuclear-to-cytoplasmic ratios of FUS–GFP intensities. EC_50_ was calculated using either CDD Vault curve fitting or Harmony 4.9 software.

### In vitro protein condensation, solidification and oxidation

For the condensation assay at different KCl concentrations, FUS–GFP proteins in storage buffer were diluted with 20 mM HEPES (pH 7.25) containing DMSO and lipoamide to yield 20 µl of the indicated concentrations of the protein and KCl, 0.3 mM DTT and 300 µM lipoamide (0.3% DMSO) and placed on a 384-well plate (781096, Greiner). Condensates were imaged on a Nikon TiE inverted microscope with a Nikon Apo ×60/1.2-NA water immersion objective using a Yokogawa CSU-X1 spinning disk head and an Andor iXon EM + DU-897 EMCCD camera

The assay to determine dilute-phase concentrations at different temperatures was performed with a newly established technique, which will be reported in detail elsewhere. In brief, the technique is based on mass and volume conservation and defined reaction volumes. We can use this method to determine accurate values for both dilute and condensed branch protein concentrations. Here, FUS–GFP phase separation was induced for a protein concentration titration in water-in-oil emulsions in a buffer containing 25 mM Tris-HCl (pH 7.4), 150 mM KCl, 1 mM DTT and the indicated concentrations of lipoamide (or DMSO as control) and imaged with a CSU-W1 (Yokogawa) spinning disk confocal system on an IX83 microscope with a UPlanSApo ×40/0.95-NA air objective, controlled via CellSens (Olympus). The dilute-phase protein concentration was derived from a linear fit to the volume of fractions of condensed-phase FUS–GFP versus the total concentrations of FUS–GFP. Temperature was controlled using a custom-made stage^81^.

For solidification assays, FUS–GFP in storage buffer was diluted in 50 mM Tris-HCl (pH 7.4) and 1 mM DTT to yield 10 μM protein, 50 mM Tris-HCl (pH 7.4), 1 mM DTT and 50 mM KCl in a volume of 20 μl in nonbinding, clear-bottom 384-well plates (781906, Greiner). Compounds (or an equal volume of DMSO) were then added for a final compound concentration of 30 μM and 0.3% DMSO. ‘Aging’ to cause fiber formation was induced by horizontal shaking at 800 rpm at room temperature, as previously described^12^. Fiber and condensate formation were analyzed by using a widefield DeltaVision Elite microscope (GE Healthcare Life Sciences) with a Plan ApoN ×60/1.4-NA oil immersion objective (Olympus) and an sCMOS camera (PCO). FRAP of FUS–GFP condensates and fibers was performed on a Nikon TiE inverted microscope with a Nikon Apo ×100/1.49-NA oil immersion objective using a Yokogawa CSU-X1 spinning disc head and an Andor iXon EM + DU-897 EMCCD camera. Regions (10 × 10 pixels) were bleached for 50 ns with a 6-mW, 405-nm laser using an Andor FRAPPA beam delivery unit and imaged for 5 min at 5 Hz. Recovery curves were derived using scripts in Fiji.

Oxidation of SFPQ was detected by change in mobility in SDS–PAGE without reducing agents. Untagged SFPQ (10 µM) in buffer (20 mM HEPES (pH 7.25) and 150 mM KCl) was incubated with H_2_O_2_ at room temperature for 30 min before subjecting to SDS–PAGE. For condensation assays of individual proteins with H_2_O_2_, SFPQ–GFP and FUS–GFP condensates were induced in buffer (20 mM HEPES (pH 7.25) and 75 mM KCl). Assays for dissolution and revival of FUS–SNAP condensates were performed in buffer (20 mM HEPES (pH 7.25) and 150 mM KCl). FUS–SNAP was labeled with SNAP-Surface Alexa Fluor 546 (New England Biolabs), and protein mixtures were oxidized with H_2_O_2_ at room temperature for 1 h before image acquisition. Proteins were imaged similar to the FUS–GFP condensates described above.

### Controlled droplet fusion using optical tweezers

Liquidity of FUS protein condensates was assessed by controlled fusion experiments using dual-trap optical tweezers, as detailed previously^12,37^. In short, for each independent fusion event, two FUS protein droplets in the presence of 300 µM lipoamide or an equivalent amount of DMSO (0.3%) as the control were trapped in each optical trap and brought into contact to initiate droplet coalescence. Fusion relaxation times were accurately recorded as changes to the laser signal as condensate material flowed into the space between the two optical traps during coalescence. The laser signal was recorded at 1 kHz, smoothed at 100 Hz and used to extract the characteristic relaxation time. After fusion was complete (as indicated by a stable laser signal), the fused droplet was discarded, and two new droplets were captured for quantifying an independent fusion event.

### Ex vivo DNA cut assays

UV microirradiation was performed as previously described^12,44^. Briefly, iPS cells expressing WT FUS–GFP were stressed by the addition of 1 mM arsenate for 1 h and treated with lipoamide or an equal volume of DMSO for 1 h. A single point in the nucleus was subject to three UV pulses as described for FRAP but at 10% laser power. GFP fluorescence was imaged at 1 Hz, and the intensity of the response was analyzed on Fiji. iPS cell MNs expressing FUS-P525L–GFP were pretreated with 20 µM lipoamide for 24 h before laser irradiation. The UV laser cutter setup used a 355-nm UV-A laser with a pulse length of <350 ps. A Zeiss α Plan-Fluar ×100/1.45-NA oil immersion objective was used, and 12 laser shots in 0.5-µm steps were administered over a 12-µm linear cut.

### Nuclear magnetic resonance for FUS–lipoamide interaction

Untagged FUS low-complexity domain (residues 1 to 163) was expressed, purified and analyzed using ^1^H-^15^N heteronuclear single quantum coherence NMR and sample conditions as previously described^82^ in the presence of 500 μM lipoamide or equivalent DMSO solvent control (1%).

### [^15^N]-Labeled lipoamide nuclear magnetic resonance

[^15^N]-Racemic (±) and (*R*)-(+)-lipoamide were synthesized from racemic and (*R*)-(+)-lipoic acid, respectively, by activating the carboxylic acid using *N*-hydroxysuccinimide and 1-ethyl-3-(3-dimethylaminopropyl) carbodiimide hydrochloride. The NHS derivative was reacted with ^15^NH_4_Cl to incorporate the [^15^N] labeling. Full details of the synthesis and subsequent biophysical validation are included in Supplementary Note 1.

For NMR quantification of [^15^N]-labeled lipoamide, ^1^H detected ^15^N edited ^1^H sensitivity enhanced HSQC NMR ((^15^N)^1^H) spectra were acquired on a 14.1T Varian Inova spectrometer equipped with a 5-mm *z*-axis gradient triple-resonance room temperature probe. Free induction decay was recorded for an acquisition time of 0.0624 s and spectral width of 8 kHz recorded over 1,000 points and a recovery delay of 1 s. Typically, 10,000 transients were collected, yielding a total experiment time of 3 h and 1 min. J coupling between the amide protons and the ^15^N in water samples was determined to be 88 Hz, and so the transfer times of 1/4 J in the INEPT portions of the pulse sequence were set to 2.6 ms. With these settings, [^15^N]ammonia or ammonium ions would not be detectable. Chemical modification of [^15^N]lipoamide (including covalent attachment to an apoenzyme) would yield a substantial change in the (^15^N)^1^H NMR spectrum. Similarly, dissolution of lipoamide in a phospholipid membrane would yield substantial peak broadening in the cell samples. We observed neither, consistent with freely diffusing lipoamide.

For optimization of the NMR measurement conditions of [^15^N]-labeled lipoamide, solvent, pH and temperature sensitivity of the primary amide proton chemical shifts were determined using dummy samples assembled from the appropriate solvent and added compounds.

Integrated NMR signal intensity is proportional to concentration if provided conditions (temperature and pH) are identical^83^. Chemical exchange^84^, expected as the amide protons in lipoamide should be labile in water, must also be accounted for. To select appropriate conditions, we determined temperature (Extended Data Fig. 3f) and pH (Extended Data Fig. 3g) sensitivity of the amide proton signal of 1 mM [^15^N]lipoamide in cell medium. Both amide protons showed chemical exchange under high-temperature, high-pH conditions, with the *trans*-amide proton affected weakly (Extended Data Fig. 3f,g). We then assessed degradation of the *trans*-amide proton over 10 h (Extended Data Fig. 3h). At 37 °C, but not 10 °C, the signal intensity decayed slowly, suggesting slow hydrolysis to form ammonia. We concluded that at 10 °C and below pH 8.6, the integrated signal from the *trans*-amide proton resonance is a good measure of [^15^N]lipoamide concentration.

For quantification of [^15^N]lipoamide cellular uptake, HeLa cells expressing FUS–GFP were grown in six-well plates to 10^6^ cells per well in DMEM supplemented with 10% fetal calf serum. To simultaneously stress and treat cells, the medium was replaced with 0.6 ml of medium supplemented with potassium arsenate and 100 μM [^15^N]-racemic (±) or (*R*)-(+)-lipoamide for 1 h at 37 °C. High concentrations of compound were used to maximize the signal. The medium was then removed and retained (medium sample), the cells were washed with ~2 ml of PBS, and the cells were removed by trypsinization with 0.3 ml of TrypLE Express (12604013, Thermo Fisher Scientific) and incubation at 37 °C for 5 min; 0.3 ml of medium was added to quench the trypsin. The resuspended cells were retained (cell sample). All samples were frozen at −80 °C. Wells were prepared for all combinations of no compound (1% DMSO control), [^15^N](±)-lipoamide or [^15^N]-(*R*)-(+)-lipoamide, with or without potassium arsenate and with or without cells.

The concentrations of [^15^N]lipoamide inside (*C*_cell_) and outside (*C*_out_) the cells were calculated from measurements of signal intensity *S* of the *trans*-amide proton of lipoamide acquired in the absence (−*cells*, sample i; Extended Data Fig. 3a) and presence (+*cells*, sample ii; Extended Data Fig. 3a) of HeLa cells, using the following equations:

$${C}_{{{\mathrm{out}}}}=\left(1-U\;\right)\frac{{c}_{{{\mathrm{add}}}}{V}_{{{\mathrm{add}}}}}{{V}_{{{\mathrm{out}}}}}$$

$${C}_{{{\mathrm{cell}}}}=U\frac{{c}_{{{\mathrm{add}}}}{V}_{{{\mathrm{add}}}}}{{V}_{1}{N}_{{{\mathrm{cell}}}}}$$

where *N*_cell_ = 10^6^, *c*_add_ = 100 μM, and *V*_add_ (added volume) = 600 μl. *V*_1_ = 4.19 × 10^−15^ m^3^ (approximating HeLa cells as spheres of radius 10^−5^ m), and *U* represents measured fractional uptake as given by

$$U=1-\frac{{S}_{+{cells}}}{{S}_{-{cells}}}.$$

To measure in vitro partitioning of [^15^N](±)-lipoamide in the FUS–GFP condensate phase, phase separation of FUS–GFP, at room temperature, was achieved by diluting 12.5 μl of protein stock at 170 μM (in salty HEPES buffer: 50 mM HEPES, 500 mM KCl, 5% glycerol (pH 7.25) and DTT 1 mM) with 247 μl of salt-free buffer containing [^15^N](±)-lipoamide (50 mM HEPES, 5% deuterium oxide, 105 μM [^15^N](±)-lipoamide and 1.05% DMSO (pH 8)), resulting in samples of 260 μl with 8 μM FUS–GFP, 100 μM [^15^N](±)-lipoamide and 25 mM KCl.

The sample was centrifuged for 10 min at 4,000*g* at room temperature, and the supernatant was kept for NMR analysis. The remaining supernatant was carefully pipetted out, without disturbing the condensate pellet, and discarded. Perpendicular view photographs of the pellet were taken. Finally, the condensate pellet was resuspended in 260 μl of buffer with the same buffer conditions as the phase-separated sample (50 mM HEPES and 25 mM KCl (pH ~7.4)).

Resuspended condensate or supernatant was loaded in deuterium oxide-matched 5-mm Shigemi tubes and analyzed by (^15^N)^1^H NMR. To achieve adequate signal to noise, the resuspended condensate was scanned for 20 h, whereas the supernatant was scanned for 4 h. The signal factor (intensity ratio between the dilute and resuspended condensate samples) was adjusted accounting for differences in sample volume and the number of scans.

The volume of condensate was calculated from perpendicular photographs (see Extended Data Fig. 4c) from the pellet radius (*a*) and inner radius of the semispherical bottom of the microcentrifuge tube (*r*):

$$V=\frac{{\rm{\pi }}}{3}{r}^{3}\left(2+\cos {\rm{\theta }}\right){\left(1-\cos {\rm{\theta }}\right)}^{2}$$

$${\rm{\theta }}={\sin }^{-1}\frac{a}{r}$$

*θ* indicates the subtended angle, as showed in Extended Data Fig. 4c.

The ratio between the concentration of lipoamide in the condensate and dilute phases (partition coefficient (*PC*)) was calculated using

$${PC}=\,\frac{{V}_{{{\mathrm{Cond}}}}+{V}_{{{\mathrm{Added}}}}}{{SF}* {V}_{{{\mathrm{Cond}}}}}$$

where *V*_Cond_ is the volume of condensate phase, *V*_Added_ is the volume added to resuspend the condensate phase (260 μl), and *SF* is the signal intensity ratio between the dilute phase and the resuspended condensate measured by NMR.

The concentrations of lipoamide in the condensate ([*L*]_Cond_) and dilute ([*L*]_Dil_) phases were calculated as

$${[L]}_{{{\mathrm{Cond}}}}=\,\frac{{[L]}_{{{\mathrm{Tot}}}}* {V}_{{{\mathrm{Tot}}}}}{{V}_{{{\mathrm{Cond}}}}+\,\frac{{V}_{{{\mathrm{Tot}}}}-{V}_{{{\mathrm{Cond}}}}}{{PC}}}\,$$

$${[L]}_{{{\mathrm{Dil}}}}=\frac{{[L]}_{{{\mathrm{Cond}}}}}{{PC}}\,$$

where [*L*]_Tot_ is the total concentration of [^15^N](±)-lipoamide, and *V*_Tot_ is the total volume of the phase-separated sample. The fraction (%) of [^15^N](±)-lipoamide signal in the condensate phase (see Extended Data Fig. 4d) was calculated as

$${{\mathrm{Lipoamide}}\; {\mathrm{condensate}}\; {\mathrm{signal}}\; {\mathrm{fraction}}}\left( \% \right)=\frac{1}{1+{SF}}* 100.$$

For derivation of these expressions, see ‘Derivation of NMR calculations’.

### Cross-linking coupled to mass spectrometry

FUS condensates were processed and analyzed essentially as described previously^85^. In short, reconstituted droplets of lysine-rich FUS-K12 or FUS-G156E were generated by low salt (80 mM KCl) and cross-linked by the addition of H12/D12 DSS (Creative Molecules) in the presence or absence of lipoamide for 30 min at 37 °C with shaking at 600 rpm. Protein samples were quenched by the addition of ammonium bicarbonate to a final concentration of 50 mM and directly evaporated to dryness. The dried protein samples were denatured in 8 M urea, reduced by the addition of 2.5 mM TCEP at 37 °C for 30 min and subsequently alkylated using 5 mM iodoacetamide at room temperature for 30 min in the dark. Samples were digested by the addition of 2% (wt/wt) trypsin (Promega) overnight at 37 °C after adding 50 mM ammonium hydrogen carbonate to a final concentration of 1 M urea. Digested peptides were separated from the solution, retained by a C18 solid-phase extraction system (SepPak Vac 1cc tC18 (50-mg cartridges, Waters)) and eluted in 50% acetonitrile and 0.1% formic acid. Dried peptides were reconstituted in 30% acetonitrile and 0.1% trifluoroacetic acid and separated by size-exclusion chromatography on a Superdex 30 increase 3.2/300 column (GE Life Sciences) to enrich for cross-linked peptides. Peptides were subsequently separated on a PepMap C18 2 µM, 50 µM × 150 mm (Thermo Fisher Scientific) column using a gradient of 5 to 35% acetonitrile for 45 min. Mass spectrometry measurements were performed on an Orbitrap Fusion Tribrid mass spectrometer (Thermo Fisher Scientific) in data-dependent acquisition mode with a cycle time of 3 s. The full scan was performed in the Orbitrap with a resolution of 120,000, a scan range of 400–1,500 *m*/*z*, an automatic gain control target of 2.0 × 10^5^ and an injection time of 50 ms. Monoisotopic precursor selection and dynamic exclusion were used for precursor selection. Only precursor charge states of 3–8 were selected for fragmentation by collision-induced dissociation using 35% activation energy. MS^2^ was performed in the ion trap in normal scan range mode with an automatic gain control target of 1.0 × 10^4^ and injection time of 35 ms. Data were searched using xQuest in ion-tag mode. Carbamidomethylation (+57.021 Da) was used as a static modification for cysteine. Cross-links were quantified relative to the condition containing no lipoamide.

### Cultured cell transfection

Transfection for gene depletion was performed with Lipofectamine 2000 (Thermo Fisher Scientific) and esiRNA oligonucleotides targeting human genes (Eupheria Biotech), as listed in Supplementary Table 1. esiRNA targeting *Renilla* luciferase was used as a negative control. The medium was replaced 5 h after transfection, and the cells were cultured for 3 days before analysis.

### Immunocytochemistry of cultured cells

HeLa or U2OS cells were fixed with 4% paraformaldehyde (PFA) in PBS at room temperature for 15 min and washed with PBS containing 30 mM glycine. After permeabilization with 0.1% Triton X-100 in PBS at 4 °C and a following wash with glycine-containing PBS, cells were blocked with 0.2% fish skin gelatin (Sigma) in PBS (blocking buffer) at room temperature for 20 min, incubated with primary antibodies in blocking buffer overnight at 4 °C, washed with blocking buffer and incubated with secondary antibodies and DAPI in blocking buffer at room temperature for 1 h. After washing with blocking buffer, the samples were stored in PBS until imaging. For detection of endogenous SFPQ, cells were fixed with cold methanol on ice for 10 min and blocked with blocking buffer at room temperature for 20 min before treatment with primary antibodies. Samples were imaged on a Zeiss LSM 700 or 880 confocal microscope with a ×40/1.2-NA water objective (Zeiss) or Opera Phenix Plus High-Content Screening System with a ×20/1.0-NA water objective (Revvity). Segmentation of cell nuclei, the cytoplasm, stress granules and mitochondria and measurement of fluorescence intensities at each segment were performed using CellProfiler. The data were then processed using KNIME to calculate the number of stress granules per cell and nuclear-to-cytoplasmic intensity ratios of stress granule proteins. For the click-crosslink lipoamide analog, intensity ratios of the click-cross-link lipoamide analog at stress granules, mitochondria and nuclei to the cytoplasm (excluding stress granules and mitochondria) intensity; inrensity ratios without cross-linking to with cross-linking and percentage of cells that had more than two stress granules.

iPS cell MNs were fixed for 15 min at room temperature in 4% PFA in PBS. Permeabilization and blocking were performed simultaneously using 0.1% Triton X-100, 1% bovine serum albumin (BSA) and 10% FBS in PBS at room temperature for 45 min. Subsequently, primary antibodies were applied overnight at 4 °C in 0.1% BSA in PBS. The cells were washed with 0.1% BSA in PBS and incubated with secondary antibodies for 1 h at room temperature. Finally, the cells were washed three times with 0.1% BSA in PBS supplemented with 0.005% Tween-20, including Hoechst or DAPI in the second washing step. Neurofilament H was used as a marker of MNs. Samples were imaged on either a CellVoyager CV7000 automated spinning disc confocal microscope (Yokogawa) with a ×40/1.3-NA water objective or a Zeiss LSM880 laser scanning confocal microscope.

The following primary antibodies were used: rabbit anti-G3BP1 (PA5-29455, Thermo Fisher Scientific), mouse anti-TOM20 (F-10, Santa Cruz), mouse anti-SPFQ (C23, MBL), mouse anti-SRSF1 (103, Invitrogen), rabbit anti-TDP-43 (80002-1-RR, Proteintech), mouse anti-SC35 (ab11826, Abcam), rabbit anti-SP100 (HPA016707, Sigma-Aldrich), mouse anti-NPM1 (FC82291, Sigma-Aldrich), rabbit anti-HP1α (2616S, Cell Signaling), mouse anti-neurofilament H (SMI-32, Millipore) and mouse anti-β3-tubulin (T5076, Sigma-Aldrich). The following secondary antibodies were used: Alexa Fluor 488-conjugated anti-mouse, Alexa Fluor 594-conjugated anti-rabbit and anti-mouse and Alexa Fluor 647-conjugated anti-rabbit and anti-mouse (Thermo Fisher Scientific).

### UV cross-linking and click reaction

HeLa cells were treated with 3 mM arsenate for 1 h, followed by 30 µM of the click-cross-link lipoamide analog for 30 min in the presence of arsenate. The cells were then irradiated with a 305-nm light-emitting diode for 10 s for cross-linking just before fixation with 4% PFA in PBS at room temperature. The fixed cells were subjected to immunostaining as described above. After staining, the cells were subjected to click reaction with 2 µM AF594-Picolyl-Azide (CLK-1296-1, Jena Bioscience) in buffer containing 100 mM HEPES (pH 7.25), 5 mM l-ascorbic acid, 0.5 mM THPTA and 0.1 mM CuSO_4_ at 37 °C for 40 min. Cells were then washed three times with 0.1% Triton X-100 in PBS to remove free dye. Imaging was performed on a CSU-W1 (Yokogawa) spinning disk confocal system on an IX83 microscope (Olympus) with a UPlanSApo ×100/1.4-NA oil objective (Olympus).

### Treatment with l-azidohomoalanine

WT HeLa cells were first washed with and cultured in methionine-free medium (21013-24, Gibco) supplemented with 10% FBS for 1 h. The medium was then replaced with complete medium or methionine-free medium supplemented with 1 mM methionine (M9625, Sigma-Aldrich) or AHA (C10102, Invitrogen) for 2 h before the cells were stressed with 1 mM arsenate for 1 h. After fixation with 4% PFA in PBS, the cells were subjected to immunostaining to label G3BP1.

### Immunoblotting

For the puromycin incorporation assay, HeLa cells were treated with 10 µM lipoamide or control 0.1% DMSO for 1 h, followed by 1 mM arsenate for an additional 1 h in the presence of lipoamide. The cells were then treated with 91.8 µM (50 µg ml^–1^) puromycin (Sigma, P8833) for 5 min before being washed with PBS and lysed with a buffer containing 50 mM Tris-HCl (pH 7.4), 150 mM NaCl, 1% NP-40, 0.1% SDS, protease inhibitors and PhosSTOP (Roche). For testing SFPQ protein levels, HeLa cells treated with AHA as described above were washed with PBS and lysed with 1% NP-40 and 0.1% SDS in PBS on ice for 15 min. The lysates were clarified by centrifugation at 20,000*g* for 10 min, separated by SDS–PAGE, transferred onto nitrocellulose membranes and subjected to immunoblotting. The following primary antibodies were used: rabbit anti-SFPQ (ARP4-572, Aviva Systems Biology), mouse anti-α-tubulin (DM1a, Sigma-Aldrich), mouse anti-puromycin (12D10, Merk Millipore) and mouse anti-GAPDH (G8795, Sigma). IRDye 800CW and IRDye680RD (LI-COR) were used as secondary antibodies to detect signal using the Odyssey platform (LI-COR).

### Time-lapse cellular imaging

Time-lapse imaging was performed at 37 °C with 5% CO_2_. iPS cell-derived MNs were treated with 20 µM lipoamide or 0.02% DMSO (control) for 1 h and then treated with 20 µM arsenite just before image acquisition. Maximum projection images were generated, and the number of FUS–GFP^+^ foci was quantified by Fiji.

### Axonal transport assays

AH-ALS1-F58 iPS cell MNs expressing FUS-P525L were treated with 2 μM compound or an equal volume of DMSO for 3 days. Longer incubation was selected to ensure penetration and action of compounds along the length of the axon channel. A concentration of 2 μM was selected as the highest concentration where there were no toxic effects on this iPS cell line (assessed qualitatively). Analysis of axonal transport of lysosomes was performed as previously described^44^. Briefly, lysosomes were labeled by the addition of 50 nM lysotracker red (Thermo Fisher Scientific) and imaged using a Leica DMI6000 inverted microscope with a ×100/1.46-NA oil immersion objective and an Andor iXON 897 EMCCD camera in an incubator chamber (37 °C, 5% CO_2_) at 3 Hz for 120 s at either the proximal or distal end of the silicone channels harboring the axons. Kymographs were generated using Fiji. Particle tracking was used to identify proportion of particles moving faster than 0.2 μm s^–1^ for five videomicrographs. Each video includes a variable population of nonmoving background particles; therefore, for each biological replicate, data were normalized to the mean proportion of moving lysosomes (>0.2 µm s^–1^) at either MFC site (proximal and distal) in the DMSO (solvent control)-treated FUS-P525L samples in Fig. 6d.

### Protein aggregation in *C. elegans*

The effect of lipoic acid on stress granule protein aggregation in vivo was analyzed using a *C. elegans* model for stress granule formation and aggregation. As previously described, fluorescent-tagged PAB-1 forms abundant stress granules and large solid aggregates during aging or following chronic stress^47,48^. RHO-1 also aggregates during aging but is not an RNA-binding or stress granule protein. Two lines were used: fluorescently tagged PAB-1 (DCD214: N2; *uqIs24*[*pmyo-2::tagrfp::pab1gene*]) and RHO-1 (DCD13: N2; *uqIs9*[*pmyo-2::rho-1::tagrfp+ptph-1::gfp*]). Each line was analyzed as described below, except DCD13, which was maintained at 20 °C.

Animals were exposed to lipoic acid in liquid culture in a 96-well plate starting from larval stage 4 (L4) in a total volume of 50 μl of S-Complete per well (100 mM NaCl, 50 mM potassium phosphate (pH 6), 10 mM potassium citrate, 3 mM MgSO_4_, 3 mM CaCl_2_, 5 μg ml^–1^ cholesterol, 50 μM EDTA, 25 μM FeSO_4_, 10 μM MnCl_2_, 10 μM ZnSO_4_ and 1 μM CuSO_4_) supplemented with heat-killed OP50 and 50 μg ml^–1^ carbenicillin. Per experiment, a minimum of nine wells each with 13 animals were treated with (*R*)-(+)- or (*S*)-(–)-lipoic acid or an equivalent volume of DMSO.

Forty-eight hours after switching L4 animals from 20 °C to 25 °C (day 2 of adulthood), extensive aggregation of fluorescently tagged PAB-1 and RHO-1 occurs in the pharyngeal muscles. After immobilization with 2 mM levamisole, aggregation was scored using a fluorescence stereo microscope (Leica M165 FC, Plan Apo ×2.0 objective). For PAB-1, aggregates occurred primarily in the terminal bulb of the pharynx, and aggregation was scored by the number of aggregates (more than ten per animal). For RHO-1, aggregates were scored in the isthmus of the pharynx, and aggregation was scored as high (>50% of the isthmus), medium (<50%) or low (no aggregation). High-magnification images were acquired with a Leica SP8 confocal microscope with an HC Plan Apo CS2 ×63/1.40-NA oil objective using a Leica HyD hybrid detector. tagRFP::PAB-1 was detected using 555 nm as excitation and an emission range from 565 to 650 nm. Representative confocal images are displayed as maximum *z*-stack projections.

### *D. melanogaster* ALS models

All fly stocks were maintained on standard cornmeal at 25 °C in a light-/dark-controlled incubator. *w*^1118^, *UAS-eGFP*, *D42-GAL4* and *OK6-Gal4* flies were obtained from Bloomington *Drosophila* Stock Center. *UAS-FUS*^WT^, *UAS-FUS*^P525L^ and *UAS-FUS*^R521C^ flies were previously described^49,86^. *UAS-TDP-43*^WT^ and *UAS-TDP-43*^M337V^ flies were provided by J. P. Taylor (St. Jude Children’s Research Hospital, Memphis, Tennessee, USA)^87^.

Tissue-specific expression of human genes was performed with the Gal4/UAS system^88^. Climbing assays were performed as previously described^86^. Briefly, flies expressing eGFP, human FUS or TDP-43 were grown in the presence or absence of lipoic acid (430 µM; ethanol was used as the vehicle control) or lipoamide (430 µM; DMSO was used as the vehicle control) and anesthetized, placed into vials and allowed to acclimate for 15 min in new vials. Feeding these compounds did not show obvious lethality or toxicity at these concentrations. For each fly genotype, the vial was knocked three times on the base on a bench, and the number of flies climbing up the vial walls was counted. The percentage of flies that climbed 4 cm in 30 s was recorded. TDP-43-expressing flies were raised at 18 °C to suppress lethality.

For immunohistochemistry of neuromuscular junctions, parent flies were crossed on food supplemented with DMSO or lipoamide, and offspring were raised on the same food. Wandering third instar larvae were dissected and subjected to immunostaining as described previously^89^. Briefly, the dissected larvae were fixed with 4% PFA in PBS at room temperature for 20 min and washed with PBS. After removing unnecessary tissues, the samples were blocked with 0.2% fish skin gelatin (Sigma) and 0.1% Triton X-100 in PBS (blocking buffer) at room temperature for 1 h, incubated with anti-HRP-Cy3 (1:200; Jackson Immunoresearch) in blocking buffer overnight at 4 °C, washed with 0.2% Triton X-100 in PBS and incubated with Alexa Fluor 488 Phalloidin (1:5,000; Thermo Fisher Scientific) at room temperature for 2 h to visualize muscles. The samples were then washed with 0.2% Triton X-100 in PBS and mounted with 70% glycerol in PBS. Synaptic boutons of muscle 4 in abdominal segments 2, 3 and 4 (A2–A4) were imaged using a Zeiss LSM 700 or 880 confocal microscope with a ×40/1.2-NA water objective (Zeiss). Numbers of synaptic boutons and satellite boutons were counted manually.

### Quantitative PCR with reverse transcription

Quantitative PCR was performed using primer sequences targeting *GAPDH* (control) and full-length *STMN2* exactly as previously described^90^.

### Thermal proteome profiling

TPP was performed as described previously^34^. Briefly, two 150-mm dishes of HeLa cells (~6 million cells per dish) were treated with 0.1% (vol/vol) DMSO (control) or 100 µM lipoamide for 1 h. At the end of incubation, one lipoamide-treated dish and one DMSO-treated dish were stressed with 1 mM arsenate for 1 h. The second set of cells served as the control (treatment with water, vehicle in which arsenate was dissolved) for only lipoamide treatment and only DMSO treatment. All incubations were performed at 37 °C with 5% CO_2_. Following incubation, cells were washed with PBS and trypsinized. Cells were collected by centrifugation at 300*g* for 3 min. The cell pellet was resuspended in PBS containing the appropriate treatment concentrations of the compounds (lipoamide, DMSO and arsenate) at a cell density of 4 × 10^6^ cells per ml. This cell suspension was split into ten 100-µl aliquots on a PCR plate and spun at 1,000*g* for 3 min, and 80 µl of supernatant (PBS) was subsequently removed. Cell aliquots were then heated to ten different temperatures (37.0, 40.4, 44.0, 46.9, 49.8, 52.9, 55.5, 58.6, 62.0 and 66.3 °C) for 3 min in a thermocycler (SureCycler 8800, Agilent) and left at room temperature for 3 min. Subsequently, cells were lysed with 30 µl of lysis buffer (PBS containing protease inhibitors, 1.12% NP-40, 2.1 mM MgCl_2_ and phosphatase inhibitors), and the PCR plates containing the cell lysate were centrifuged at 1,000*g* for 5 min to remove cell debris. Next, heat-induced protein aggregates were removed from the cleared supernatant by passing then through a 0.45-µm, 96-well filter plate (Millipore) at 500*g* for 5 min. Equal volumes of the flow-through and 2× sample buffer (180 mM Tris-HCl (pH 6.8), 4% SDS, 20% glycerol and 0.1 g of bromophenol blue) were mixed and stored in –20 °C until use for mass spectrometry sample preparation. Protein digestion, peptide labeling and mass spectrometry-based proteomics were performed as previously described^35^.

Abundance and thermal stability scores for every protein were calculated as described previously^33,91^. Briefly, the ratio of the normalized tandem mass tag reporter ion intensity in each treatment (only lipoamide, only arsenate, lipoamide and arsenate) and the control (only DMSO) was calculated for each temperature. The abundance score for each protein was calculated as the average log_2_ (fold change) (FC) at the two lowest temperatures:

$${{\mathrm{Abundance}}\; {\mathrm{score}}}=\frac{{\log }_{2}{{{\mathrm{FC}}}}_{37.0^\circ {\mathrm{C}}}+{\log }_{2}{{{\mathrm{FC}}}}_{40.4^\circ {\mathrm{C}}}}{2}.$$

The thermal stability score for each protein was computed by subtracting the abundance score from the log_2_ (fold change) values of all temperatures and then summing the resulting fold changes (requiring that there were at least ten data points to calculate this score):

$${{\mathrm{Thermal}}\; {\mathrm{stability}}\; {\mathrm{score}}}=\sum _{T}\left({\log }_{2}{{{\mathrm{FC}}}}_{T}-{{\mathrm{abundanace}}\; {\mathrm{score}}}\right),$$

where *T* is the ten temperatures. Both the abundance and the thermal stability scores were transformed into a *z* distribution by subtracting the mean and dividing by the standard deviation. The significance of the abundance and thermal stability scores was further assessed using limma^92^ (the two scores were weighted for the number of temperatures in which a protein was identified), followed by FDR estimation using the fdrtool package^93^. Proteins with a | *z* score | of >1.5 and an FDR of <0.05 were considered significant changes for the IDR analyses. Thermal stability scores indicated in the ‘Results’ are those in cells treated with lipoamide and arsenate.

### Bioinformatics

Positions of amino acid sequences with disordered tendency were visualized using IUPred3 (https://iupred.elte.hu/). The lengths of IDRs in each protein were estimated using d2p2 database (https://d2p2.pro/)^94^. An IDR is defined as a region that is regarded as being disordered in more than 75% of all predictors in the database as well as with more than ten successive disordered amino acids. The proportions of IDRs to the whole protein amino acid length were calculated. Enrichment of individual amino acids in IDRs was calculated using Composition Profiler (http://www.cprofiler.org/)^95^. IDRs of all proteins detected by TPP were used as a background. Positive and negative scores indicate enrichment and depletion of each amino acid compared to the background, respectively.

### Statistical analysis

Statistical analyses were performed using R statistical software or GraphPad Prism. The statistical details (*P* value, number of samples and statistical test used) are specified in the figure panels or legends. A *P* value below 0.05 was considered statistically significant.

### Derivation of NMR calculations

For calculations to estimate the intracellular concentration of lipoamide, cellular uptake was measured by comparing the signal intensity *S* of the *trans*-amide proton of [^15^N](±)-lipoamide acquired in the absence (−*cells*, sample i; Extended Data Fig. 3a) and presence (+*cells*, sample ii; Extended Data Fig. 2a) of HeLa cells. The measured fractional uptake ${\boldsymbol{U}}$ was given by

$$U=1-\frac{{S}_{+{cells}}}{{S}_{-{cells}}}.$$

The quantity (moles) of lipoamide added (${{\mathrm{add}}}$) becomes distributed between the intracellular (${{\mathrm{cell}}}$) and extracellular (${{\mathrm{out}}}$) environments following uptake. This can be expressed in terms of concentrations $c$ and volumes $V$:

$${c}_{{{\mathrm{add}}}}{V}_{{{\mathrm{add}}}}={c}_{{{\mathrm{cell}}}}{V}_{{{\mathrm{cell}}}}+{c}_{{{\mathrm{out}}}}{V}_{{{\mathrm{out}}}}.$$

The total volume of cells is given by ${V}_{{{\mathrm{cell}}}}={V}_{1}{N}_{{{\mathrm{cell}}}}$, where ${V}_{1}$ is the volume of a single cell, and ${N}_{{{\mathrm{cell}}}}$ is the number of cells. ${V}_{{{\mathrm{cell}}}}\ll \,{V}_{{{\mathrm{add}}}}$, so we assume ${V}_{{{\mathrm{add}}}}={V}_{{{\mathrm{out}}}}$. The fractional uptake can also be written in terms of these concentrations and volumes:

$$U=1-\frac{{c}_{{{\mathrm{out}}}}{V}_{{{\mathrm{out}}}}}{{c}_{{{\mathrm{add}}}}{V}_{{{\mathrm{add}}}}}.$$

Rearrangement yields expressions for the concentration inside and outside the cell in terms of the quantity of lipoamide added and the measured fractional uptake $U$:

$${C}_{{{\mathrm{out}}}}=\left(1-U\;\right)\frac{{c}_{{{\mathrm{add}}}}{V}_{{{\mathrm{add}}}}}{{V}_{{{\mathrm{out}}}}}$$

$${C}_{{{\mathrm{cell}}}}=U\frac{{c}_{{{\mathrm{add}}}}{V}_{{{\mathrm{add}}}}}{{V}_{1}{N}_{{{\mathrm{cell}}}}}$$

We approximate HeLa cells as spheres of radius 10^−5^ m, ${V}_{1}=$ 4.19 × 10^−15^ m^3^. In our experiment, ${N}_{{{\mathrm{cell}}}}=$ 10^6^, ${c}_{{{\mathrm{add}}}}=$ 100 μM, and ${V}_{{{\mathrm{add}}}}=$ 600 μl, respectively.

We saw no evidence for peak broadening, which would be associated with dissolution in a phospholipid membrane; however, in principle, the lost signal intensity on uptake could be attributed to uptake into membranes rather than the cytoplasm. Calculation suggests that this is implausible. The number of phospholipid molecules in the plasma membrane can be estimated from the footprint of each lipid molecule ${A}_{L}=$ 0.5 nm^2^. Assuming a spherical cell, the surface area of a single cell is ${A}_{1}=$ 1.3 × 10^−9^ m^2^. Therefore, the total number of phospholipids on the cell surface is given by ${N}_{L}=\frac{{A}_{1}{N}_{{{\mathrm{cell}}}}}{{A}_{L}}$. The number of lipoamide molecules taken up by cells is given by ${N}_{{{\mathrm{uptake}}}}={c}_{{{\mathrm{cell}}}}{V}_{{{\mathrm{cell}}}}{N}_{{\mathrm{A}}}$, where *N*_A_ is Avogadro’s number. The ratio $R$ of lipoamide to lipid molecules is given by

$$R=\frac{{N}_{{{\mathrm{uptake}}}}}{{N}_{L}}=\frac{{A}_{L}{N}_{{\mathrm{A}}}U{c}_{{{\mathrm{add}}}}{V}_{{{\mathrm{add}}}}}{{A}_{1}{N}_{{{\mathrm{cell}}}}}.$$

For the mean experimentally observed $U=$ 0.35 (that is, 35% uptake), then we expect $R=$ 4.9, that is, 4.9 lipoamide molecules per plasma membrane lipid molecule. The plasma membrane is not the only membrane in the cell. Even if it makes up 10% of total phospholipid, there would need to be approximately one lipoamide molecule per two phospholipid molecules.

For estimation of the partitioning of lipoamide in FUS–GFP droplets, the following manipulations follow standard chemical methods. We define the partition coefficient (*PC*) as the ratio between the concentration in the condensate ([*L*]_Cond_) and dilute ([*L*]_Dil_) phases:

$${PC}=\frac{{[L]}_{{{\mathrm{Cond}}}}}{{[L]}_{{{\mathrm{Dil}}}}}.$$

We know that the total [^15^N](±)-lipoamide amount distributes between the phases as follows:

$${\left[L\right]}_{{{\mathrm{Tot}}}}{V}_{{{\mathrm{Tot}}}}={\left[L\right]}_{{{\mathrm{Cond}}}}{V}_{{{\mathrm{Cond}}}}{\boldsymbol{+}}{\left[L\right]}_{{{\mathrm{Dil}}}}{V}_{{{\mathrm{Dil}}}},$$

where ${[L]}_{{{\mathrm{Tot}}}}$ (total concentration of lipoamide) = 100 μM, ${V}_{{{\mathrm{Tot}}}}$ (total volume) = 260 μl, and ${V}_{{{\mathrm{Cond}}}}$ and ${V}_{{{\mathrm{Dil}}}}$ are the volumes of the condensate and dilute phases, respectively.

Using NMR, we measured the intensity ratio, or signal factor (*SF*), between the dilute phase and the resuspended condensate phase (compensating for differences in NMR sample volume),

$${SF}=\frac{{[L]}_{{{\mathrm{Dil}}}}}{{[L]}_{{{\mathrm{RCond}}}}},$$

where ${[L]}_{{{\mathrm{RCond}}}}$ is the concentration of lipoamide in the condensate once resuspended with a volume ${V}_{{{\mathrm{Added}}}}$:

$${[L]}_{{{\mathrm{RCond}}}}=\frac{{V}_{{{\mathrm{Cond}}}}{[L]}_{{{\mathrm{Cond}}}}}{{V}_{{{\mathrm{Cond}}}}+\,{V}_{{{\mathrm{Added}}}}}.$$

Combining the previous two equations, we can define ${[L]}_{{{\mathrm{Dil}}}}$ as

$${[L]}_{{{\mathrm{Dil}}}}={SF}\frac{{V}_{{{\mathrm{Cond}}}}{\left[L\right]}_{{{\mathrm{Cond}}}}}{{V}_{{{\mathrm{Cond}}}}+\,{V}_{{{\mathrm{Added}}}}},$$

and we substitute ${[L]}_{{{\mathrm{Dil}}}}$ in the initial equation to obtain

$${PC}=\,\frac{{[L]}_{{{\mathrm{Cond}}}}}{\frac{{SF}{V}_{{{\mathrm{Cond}}}}{[L]}_{{{\mathrm{Cond}}}}}{{V}_{{{\mathrm{Cond}}}}+{V}_{{{\mathrm{Added}}}}}}=\,\frac{{V}_{{{\mathrm{Cond}}}}+{V}_{{{\mathrm{Added}}}}}{{SF}{V}_{{{\mathrm{Cond}}}}},$$

where *V*_Cond_ is the volume of condensate phase measured (main text methods), ${V}_{{{\mathrm{Added}}}}$ is the volume added to resuspend the condensate phase for NMR analysis (260 μl), and *SF* is the signal intensity ratio between the dilute phase and the resuspended condensate measured by NMR and defined above.

Additionally, we can calculate the concentration of lipoamide in the condensate phase by rearranging the first equation and substituting ${[L]}_{{{\mathrm{Dil}}}}$ in the second, also considering that ${V}_{{{\mathrm{Dil}}}}={V}_{{{\mathrm{Tot}}}}-{V}_{{{\mathrm{Cond}}}}$:

$${[L]}_{{{\mathrm{Cond}}}}=\,\frac{{[L]}_{{{\mathrm{Tot}}}}\,{V}_{{{\mathrm{Tot}}}}}{{V}_{{{\mathrm{Cond}}}}+\,\frac{{V}_{{{\mathrm{Tot}}}}-{V}_{{{\mathrm{Cond}}}}}{{PC}}}.$$

From this last expression and using the obtained partition coefficient, we can also calculate the concentration of lipoamide in the dilute phase. From the expression of *SF*, we can calculate the fraction (%) of [^15^N]±-lipoamide signal in the resuspended pellet (condensate phase) as (Extended Data Fig. 4d)

$$\begin{array}{l}{{\mathrm{Lipoamide}}\; {\mathrm{condensate}}\; {\mathrm{signal}}}\\=\,\frac\displaystyle{{S}_{{{\mathrm{RCond}}}}}{{S}_{{{\mathrm{RCond}}}}+\,{S}_{{{\mathrm{Dil}}}}}=\frac\displaystyle{{S}_{{{\mathrm{RCond}}}}}{{S}_{{{\mathrm{RCond}}}}+{SF}{\;S}_{{{\mathrm{RCond}}}}}=\frac\displaystyle{1}{1+{SF}}\end{array},$$

where *S*_RCond_ is the signal of the resuspended condensate, and *S*_Dil_ is the signal of the supernatant.

### Reporting summary

Further information on research design is available in the Nature Portfolio Reporting Summary linked to this article.

## Online content

Any methods, additional references, Nature Portfolio reporting summaries, source data, extended data, supplementary information, acknowledgements, peer review information; details of author contributions and competing interests; and statements of data and code availability are available at 10.1038/s41589-025-01893-5.

## Supplementary information

Supplementary Information Supplementary Note 1.

Reporting Summary

Supplementary Table 1 Genes tested in the RNAi knockdown screen.

## Source data

Source Data Fig. 1 Statistical source data.

Source Data Fig. 2 Statistical source data.

Source Data Fig. 3 Statistical source data.

Source Data Fig. 4 Statistical source data.

Source Data Fig. 5 Statistical source data.

Source Data Fig. 6 Statistical source data.

Source Data Extended Data Fig. 1 Statistical source data.

Source Data Extended Data Fig. 2 Statistical source data.

Source Data Extended Data Fig. 3 Statistical source data.

Source Data Extended Data Fig. 4 Statistical source data.

Source Data Extended Data Fig. 5 Statistical source data.

Source Data Extended Data Fig. 6 Statistical source data.

Source Data Extended Data Fig. 7 Data.

Source Data Extended Data Fig. 7 Gel image.

Source Data Extended Data Fig. 8 Statistical source data.

Source Data Extended Data Fig. 9 Statistical source data.

## Figures and Tables

**Extended Data Table 1:**
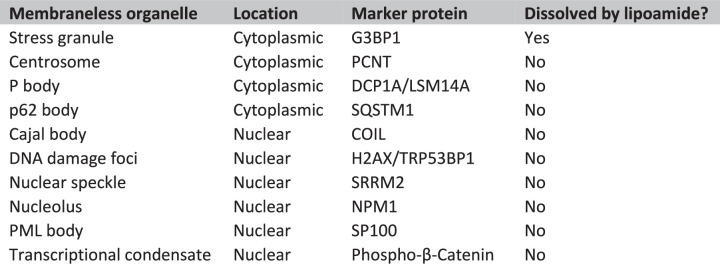
Lipoamide effect on multiple membraneless organelles

## Data Availability

Data supporting the findings of this work are provided in the manuscript and its source data. The mass spectrometry proteomics data have been deposited to the ProteomeXchange Consortium via the PRIDE^96^ partner repository with the dataset identifiers PXD039670 (for the cross-linking assay) and PXD039501 (for the TPP assay). Source data are provided with this paper.
