# Supplementary material for: Small-molecule dissolution of stress granules by redox modulation benefits ALS models
Source: Nat Chem Biol. 2025 May 14;21(10):1577–88. doi: 10.1038/s41589-025-01893-5 (PMC12463676; doi:10.1038/s41589-025-01893-5)
Supplement: Supplementary file 1 — Supplementary Note 1. [file 41589_2025_1893_MOESM1_ESM.pdf]

nature chemical biology

Supplementary information https://doi.org/10.1038/s41589-025-01893-5

Small-molecule dissolution of stress granules by redox modulation benefits ALS models

In the format provided by the authors and unedited

Supplementary Note 1

Synthesis of ^{15}N-labelled racemic (±)-lipoamide

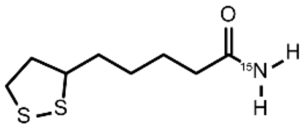

Racemic (±)-lipoic acid (1.08 g, 5.23 mmol), *N*-hydroxysuccinimide (660 mg, 5.73 mmol) and 1-ethyl-3-(3-dimethylaminopropyl) carbodiimide hydrochloride (1.10 g, 5.74 mmol) were dissolved in dry DMF (20 mL) under argon at 25 °C. Then, the mixture was stirred for 4 h, and it was diluted with ethyl acetate (100 mL). The mixture was washed with water (100 mL), saturated aqueous $NaHCO_3$ (100 mL) and again with water (2 × 100 mL). The organic layer was dried over anhydrous $MgSO_4$, filtered and the solvent was removed from the filtrate by rotary evaporation to obtain the crude racemic (±)-lipoic acid NHS ester. The crude NHS ester obtained from the above process was dissolved in dry DCM (20 mL) at 25 °C. Then, $^{15}NH_4Cl$ (500 mg, 9.18 mmol) and triethylamine (1.10 mL, 7.89 mmol) were added to the mixture. The mixture was stirred for 20 h. The solution was diluted with DCM (100 mL), washed with water (100 mL), saturated aqueous $NaHCO_3$ (100 mL) and again with water (2 × 100 mL). The organic layer was dried over anhydrous $MgSO_4$, filtered and the solvent was removed from the filtrate $MgSO_4$ by rotary evaporation to give crude ^{15}N-labelled racemic (±)-lipoamide, which was further purified by silica gel flash column chromatography (DCM/methanol, 30:1). The solvent was removed by rotary evaporation and [^{15}N](±)-lipoamide was obtained as a yellow solid (554 mg, 51% yield).

TLC R_f (DCM/methanol, 20:1) = 0.60; **MP** 130 °C (lit[1] MP 129–131 °C for unlabelled racemic (±)-lipoamide); **^{1}H NMR** (400 MHz, $CDCl_3$): δ 5.52 (d, *J* = 88.0 Hz, 1H, NH_{cis}), 5.44 (d, *J* = 88.4 Hz, 1H, NH_{trans}), 3.57 (m, 1H, SSC*H*), 3.21 – 3.08 (m, 2H, SSCH_2), 2.50 – 2.42 (m, 1H, $SSCH_2CH_{trans}$), 2.23 (t, *J* = 8.0 Hz, 2H, CH_2CONH_2), 1.95 – 1.87 (m, 1H, $SSCH_2CH_{cis}$), 1.77 – 1.60 (m, 4H), 1.56 – 1.42 (m, 2H, $CH_2CH_2CH_2CONH_2$); **^{13}C NMR** (151 MHz, $CDCl_3$) δ 175.2 (d, *J* = 13.6 Hz, *C*ONH_2), 56.5 (SS*C*H), 40.4 ($SSCH_2CH_2$), 38.6 ($SSCH_2$), 35.7 (CH_2CONH_2), 34.8 ($CH_2CH_2CONH_2$), 29.0 ($CH_2CH_2CH_2CONH_2$), 25.3 ($CH_2CH_2CH_2CH_2CONH_2$); **^{15}N NMR** (61 MHz, $CDCl_3$) δ = 101.8 (t, *J* = 88.6 MHz, CO*N*H_2); **HRMS** (ESI^+) *m/z* [MNa+] calcd for $C_8H_{15}O^{15}NS_2Na$, 229.0458; found 229.0459; **IR (neat/cm^{-1})**: 3352m, 3176m, 2937m, 1650s, 1629m, 1464m, 1413s.

^{15}N-labelled racemic (±)-lipoamide ^{1}H NMR spectrum (400 MHz, $CDCl_3$)

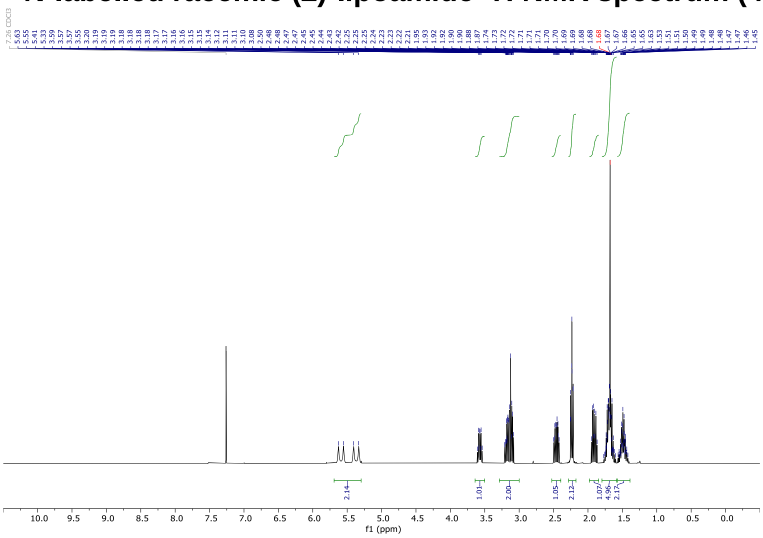

^{15}N-labelled racemic (±)-lipoamide ^{13}C NMR spectrum (151 MHz, $CDCl_3$)

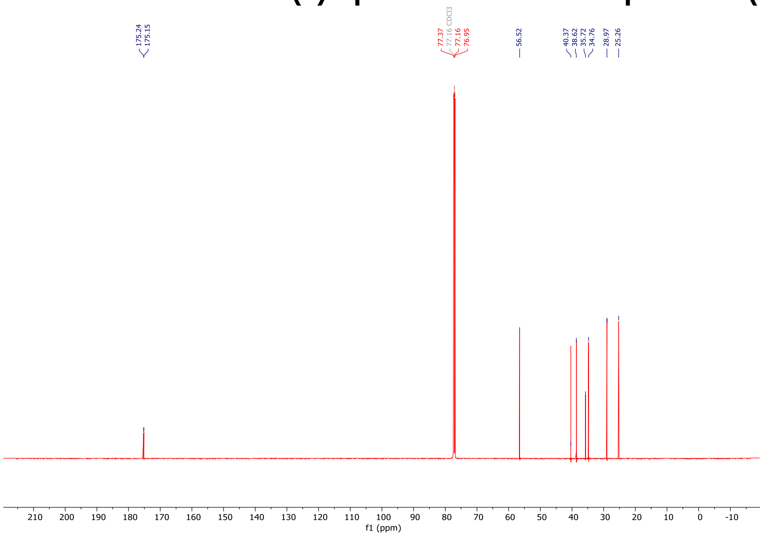

^{15}N-labelled racemic (±)-lipoamide ^{15}N NMR spectrum (61MHz, $CDCl_3$)

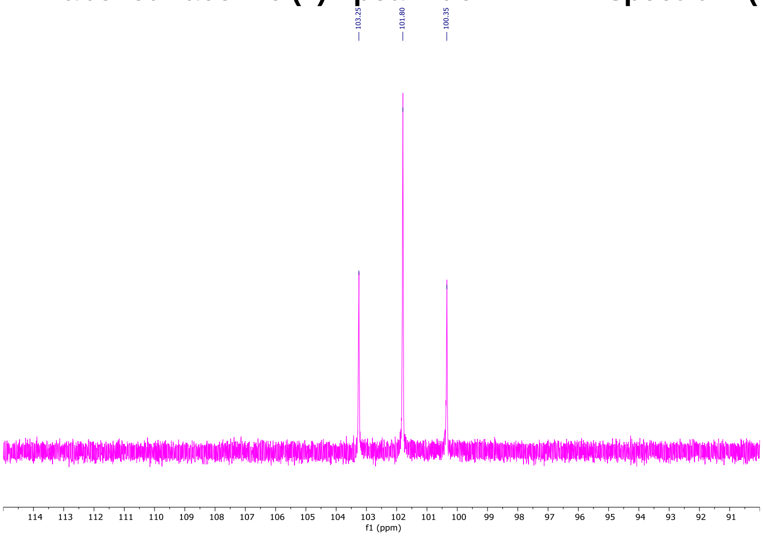

^{15}N-labelled racemic (±)-lipoamide HRMS (ESI$^+$)

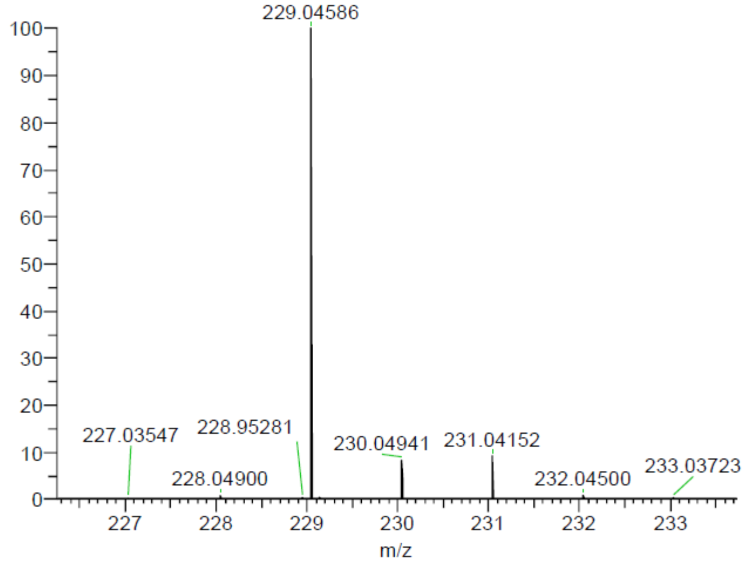

Formula (M)	Ion Formula	m/z	m/z (Calc)	Diff (ppm)
C8 H15 O [15]N S2	C8 H15 O [15]N S2 Na	229.04585	229.04576	0.4

HRMS data suggested that the ^{15}N labelling efficacy is at ~99%.

DFT calculations for the assignment of the ^{15}N-labelled racemic (±)-lipoamide ^{1}H NMR

Density functional theory (DFT) calculations were performed to confirm the assignments of proton resonances in the ^{1}H NMR spectrum obtained from ^{15}N-labelled racemic (±)-lipoamide. An optimised structure for the [^{15}N](±)-lipoamide was generated using Gaussian098 from which shielding tensors were calculated, enabling isotropic and anisotropic components to be determined. DFT calculations used the B3LYP density functional with the 6-31G(d) basis set.[2]

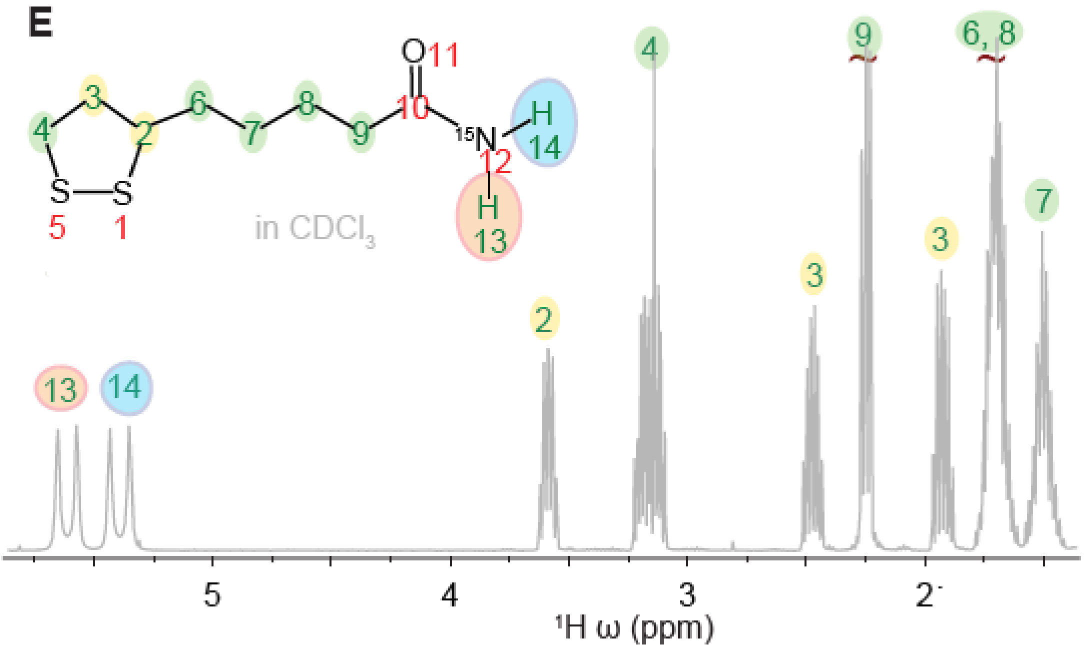

DFT calculations suggest that the *cis*-amide proton 13 (*cis* to the alkyl chain) should have a larger chemical shift than the *trans*-amide proton 14 (*trans* to the alkyl chain), and that the proton bound to carbon 3 closest in proximity to the proton bound to carbon 2 should have a larger chemical shift than the other proton on carbon 3.

Synthesis of ^{15}N-labelled (*R*)-(+)-lipoamide

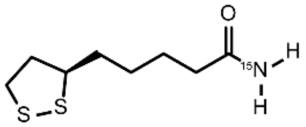

(*R*)-(+)-Lipoic acid (1.08 g, 5.23 mmol), *N*-hydroxysuccinimide (660 mg, 5.73 mmol) and 1-ethyl-3-(3-dimethylaminopropyl)carbodiimide hydrochloride (1.10 g, 5.74 mmol) were dissolved in DMF (20 mL) under argon at 25 °C. Then, the mixture was stirred for 4 h, and it was diluted with ethyl acetate (100 mL). The mixture was washed with water (100 mL), saturated aqueous $NaHCO_3$ (100 mL) and again with water (2 × 100 mL). The organic layer was dried over anhydrous $MgSO_4$, filtered and the solvent was removed from the filtrate by rotary evaporation to obtain the crude (*R*)-(+)-lipoic acid NHS ester. This crude NHS ester obtained from the above process was dissolved in dry DCM (20 mL) at 25 °C. Then, $^{15}NH_4Cl$ (500 mg, 9.18 mmol) and triethylamine (1.10 mL, 7.89 mmol) were added to the mixture. The mixture was stirred for 20 h. The solution was diluted with DCM (100 mL), washed with water (100 mL), saturated aqueous $NaHCO_3$ (100 mL) and again with water (2 × 100 mL). The organic layer was dried over anhydrous $MgSO_4$, filtered and the solvent was removed from the filtrate by rotary evaporation to give crude ^{15}N-labelled (*R*)-(+)-lipoamide, which was further purified by silica gel flash column chromatography (DCM/methanol, 30:1). The solvent was removed by rotary evaporation and [^{15}N]-(*R*)-(+)-lipoamide was obtained as yellow solid (555 mg, 51% yield).

TLC R_f (DCM/methanol, 20:1) = 0.60; **MP** 131.5 °C; **^{1}H NMR** (400 MHz, $CDCl_3$) δ 5.60 (d, *J* = 88.0 Hz, 1H, NH_{cis}), 5.48 (d, *J* = 88.8 Hz, 1H, NH_{trans}), 3.57 (m, 1H, SSC*H*), 3.21 – 3.08 (m, 2H, SSCH_2), 2.49 – 2.42 (m, 1H, SSCH2CH_{trans}), 2.23 (t, *J* = 8.0 Hz, 2H, CH_2CONH_2), 1.95 – 1.86 (m, 1H, SSCH_2CH_{cis}), 1.73 – 1.63 (m, 4H), 1.54 – 1.43 (m, 2H, CH_2CH_2CH_2CONH_2); **^{13}C NMR** (151 MHz, $CDCl_3$) δ = 175.3 (d, *J* = 13.6 Hz, *C*ONH_2), 56.5 (SS*C*H), 40.4 (SSCH_2*C*H_2), 38.6 (SS*C*H_2), 35.7 (*C*H_2CONH_2), 34.7 (*C*H_2CH_2CONH_2), 29.0 (*C*H_2CH_2CH_2CONH_2), 25.3 (*C*H_2CH_2CH_2CH_2CONH_2); **^{15}N NMR** (61 MHz, $CDCl_3$) δ = 102.1 (t, *J* = 88.7 MHz, CO*N*H_2); **HRMS** (ESI^+) *m/z* [M+] calcd for $C_8H_{15}O^{15}NS_2$, 207.0638; found 207.0637; **IR (neat/cm^{-1})**: 3343m, 3178m, 2937w, 1653s, 1631s, 1462w, 1413s; **Optical rotation**: $[\alpha]_D^{25}$ +105.6 (*c*=2.0, $CHCl_3$).

^{15}N-labelled (*R*)-(+)-lipoamide ^{1}H NMR spectrum (400 MHz, $CDCl_3$)

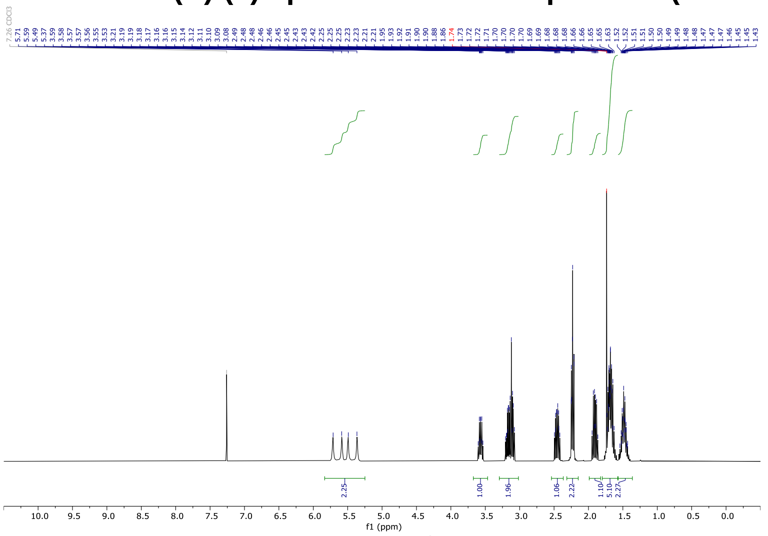

^{15}N-labelled (*R*)-(+)-lipoamide ^{13}C NMR spectrum (151 MHz, $CDCl_3$)

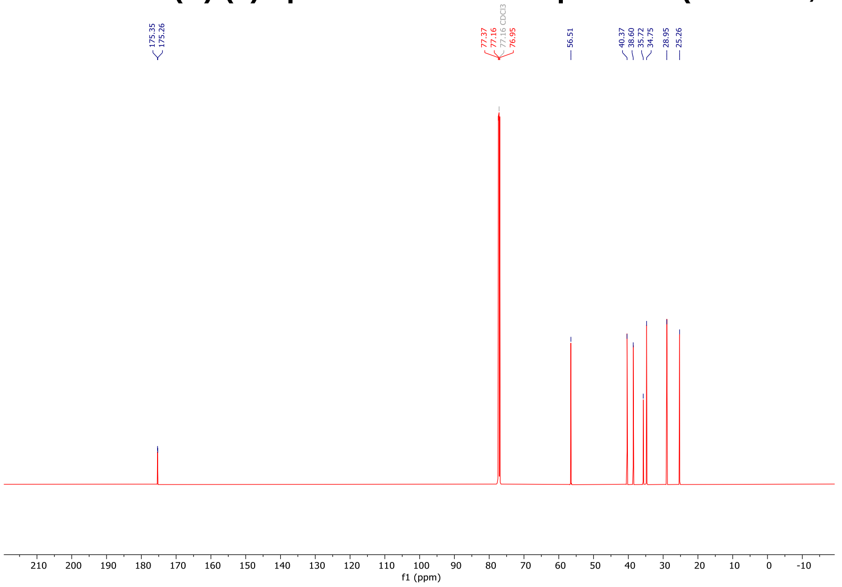

^{15}N-labelled (*R*)-(+)-lipoamide ^{15}N NMR spectrum (61MHz, $CDCl_3$)

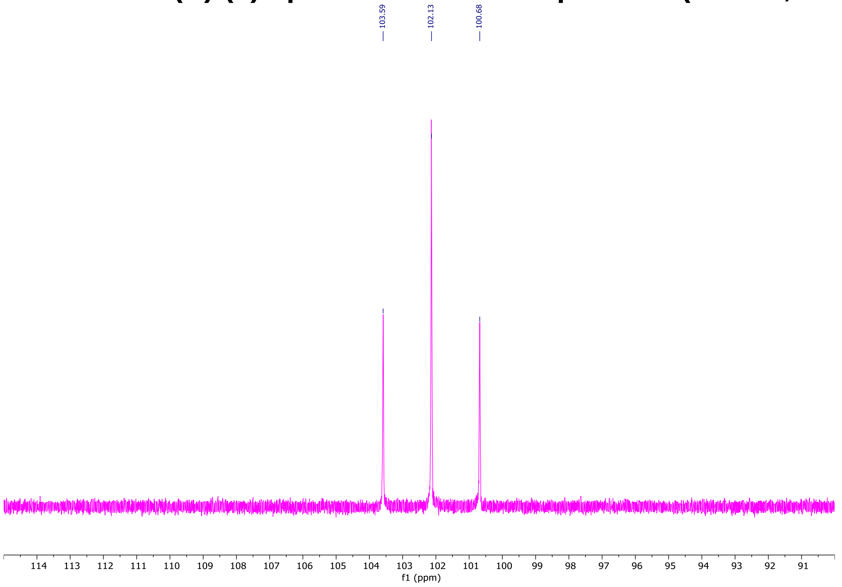

^{15}N-labelled (*R*)-(+)-lipoamide HRMS (ESI$^+$)

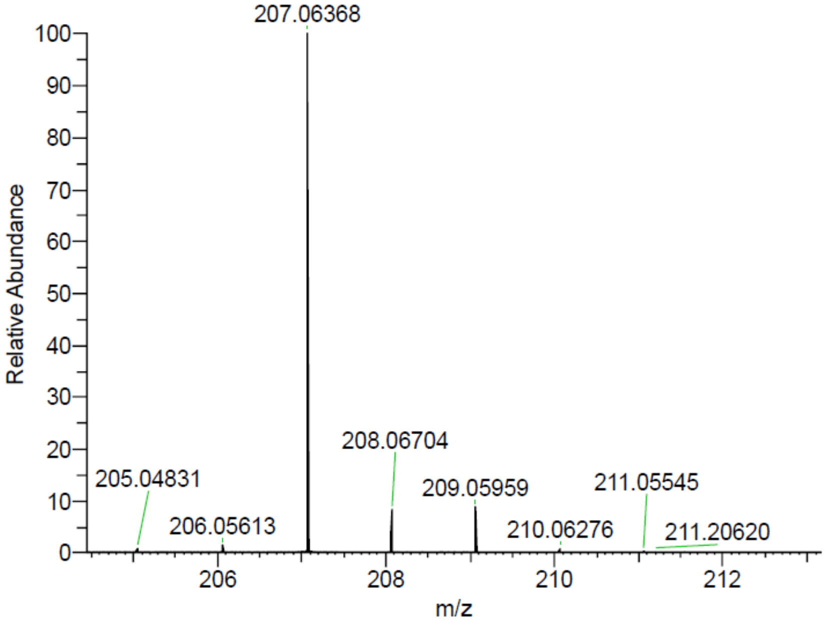

Formula (M)	Ion Formula	m/z	m/z (Calc)	Diff (ppm)
C8 H15 O [15]N S2	C8 H15 O [15]N S2	207.06368	207.06382	-1.38

HRMS data suggested that the ^{15}N labelling efficacy is at ~99%.

References

(1) Reed, L. J.; Koike, M.; Levitch, M. E.; Leach, F. R. Studies on the Nature and Reactions of Protein-Bound Lipoic Acid. *J. Biol. Chem.* **1958**, *232* (1), 143–158. https://doi.org/10.1016/S0021-9258(18)70382-7.

(2) Karunanithy, G.; Cnossen, A.; Müller, H.; Peeks, M. D.; Rees, N. H.; Claridge, T. D. W.; Anderson, H. L.; Baldwin, A. J. Harnessing NMR Relaxation Interference Effects to Characterise Supramolecular Assemblies. *Chem. Commun.* **2016**, *52* (47), 7450–7453. https://doi.org/10.1039/C6CC02544G.
