# Supplementary material for: Small-molecule dissolution of stress granules by redox modulation benefits ALS models
Source: Nat Chem Biol. 2025 May 14;21(10):1577–88. doi: 10.1038/s41589-025-01893-5 (PMC12463676; doi:10.1038/s41589-025-01893-5)
Supplement: Supplementary file 2 — Reporting Summary [file 41589_2025_1893_MOESM2_ESM.pdf]

nature portfolio

Corresponding author(s): Richard Wheeler, Anthony Hyman

Last updated by author(s): Jan 25, 2025

Reporting Summary

Nature Portfolio wishes to improve the reproducibility of the work that we publish. This form provides structure for consistency and transparency in reporting. For further information on Nature Portfolio policies, see our Editorial Policies and the Editorial Policy Checklist.

Statistics

For all statistical analyses, confirm that the following items are present in the figure legend, table legend, main text, or Methods section.

n/a	Confirmed	
☐	☒	The exact sample size (*n*) for each experimental group/condition, given as a discrete number and unit of measurement
☐	☒	A statement on whether measurements were taken from distinct samples or whether the same sample was measured repeatedly
☐	☒	The statistical test(s) used AND whether they are one- or two-sided<br>*Only common tests should be described solely by name; describe more complex techniques in the Methods section.*
☒	☐	A description of all covariates tested
☒	☐	A description of any assumptions or corrections, such as tests of normality and adjustment for multiple comparisons
☐	☒	A full description of the statistical parameters including central tendency (e.g. means) or other basic estimates (e.g. regression coefficient) AND variation (e.g. standard deviation) or associated estimates of uncertainty (e.g. confidence intervals)
☐	☒	For null hypothesis testing, the test statistic (e.g. *F*, *t*, *r*) with confidence intervals, effect sizes, degrees of freedom and *P* value noted<br>*Give P values as exact values whenever suitable.*
☒	☐	For Bayesian analysis, information on the choice of priors and Markov chain Monte Carlo settings
☒	☐	For hierarchical and complex designs, identification of the appropriate level for tests and full reporting of outcomes
☒	☐	Estimates of effect sizes (e.g. Cohen's *d*, Pearson's *r*), indicating how they were calculated

Our web collection on statistics for biologists contains articles on many of the points above.

Software and code

Policy information about availability of computer code

Data collection	For microscopy, Cell Voyager Measurement System (Yokogawa CV7000); ZEN 14.0.25.201 (Zeiss confocal microscopes); Andor iQ 3.6 (Yokogawa CSU-X1); CellSens 4.1 (CSU-W1); softWoRx 7.2.0 (Deltavision Elite). For bioinformatics analysis, IUPred3 (https://iupred3.elte.hu/); d2p2 database (https://d2p2.pro/); Composition Profiler (v1.2 http://www.cprofiler.org/).
Data analysis	For crosslinking mass spectrometry, xQuest (v2.1). For quantiative image analysis, CellProfiler (4.2.4); KNIME (4.7.0); FIJI (1.5.X); Harmony (4.9). For thermal proteome profiling, limma (3.20); fdrtool (1.2.15). For statistics, R statistical software (4.0.3); GraphPad Prism (9.3.0).

For manuscripts utilizing custom algorithms or software that are central to the research but not yet described in published literature, software must be made available to editors and reviewers. We strongly encourage code deposition in a community repository (e.g. GitHub). See the Nature Portfolio guidelines for submitting code & software for further information.

Data

Policy information about availability of data

All manuscripts must include a data availability statement. This statement should provide the following information, where applicable:

- Accession codes, unique identifiers, or web links for publicly available datasets
- A description of any restrictions on data availability
- For clinical datasets or third party data, please ensure that the statement adheres to our policy

Proteomics data is available at PRIDE with the dataset identifiers PXD039670 (crosslinking) and PXD039501 (TPP).

Research involving human participants, their data, or biological material

Policy information about studies with human participants or human data. See also policy information about sex, gender (identity/presentation), and sexual orientation and race, ethnicity and racism.

Reporting on sex and gender	N/A
Reporting on race, ethnicity, or other socially relevant groupings	N/A
Population characteristics	N/A
Recruitment	N/A
Ethics oversight	N/A

Note that full information on the approval of the study protocol must also be provided in the manuscript.

Field-specific reporting

Please select the one below that is the best fit for your research. If you are not sure, read the appropriate sections before making your selection.

☒ Life sciences ☐ Behavioural & social sciences ☐ Ecological, evolutionary & environmental sciences

For a reference copy of the document with all sections, see nature.com/documents/nr-reporting-summary-flat.pdf

Life sciences study design

All studies must disclose on these points even when the disclosure is negative.

Sample size	All cell biological, in vitro biochemical, mass spectrometry and NMR experiments were designed with sample sizes based on prevailing best practice from the literature, such as: Woodruff et al, Cell 2017; Beutel et al, Cell, 2019; Sridharan et al, Nat Commun 2019. Sample size varies assay to assay, in practice >= 2 replicates and individually specified in the text.
Data exclusions	There was no data exclusion.
Replication	The action of lipoamide on stress granules was verified through re-purchase and independent synthesis of N15 lipoamide in 4 separate laboratories. Replicability of other assays was confirmed by consistent behaviour of multiple independent replicates, as in Sample size above, in practice >= 2 replicates.
Randomization	There were not experimental groups requiring randomisation. All assays involved defined reagents: clonal cell lines, purified proteins, inbred lab strains, etc.
Blinding	Full double blind analysis was used for the axonal transport assays. Data analysis was, where possible, carried out single blinded to the sample identity but was not enforced. Experimental procedures were not generally blinded due to sample to sample differences in handling.

Reporting for specific materials, systems and methods

We require information from authors about some types of materials, experimental systems and methods used in many studies. Here, indicate whether each material, system or method listed is relevant to your study. If you are not sure if a list item applies to your research, read the appropriate section before selecting a response.

Materials & experimental systems

n/a	Involved in the study	
☐	☒	Antibodies
☐	☒	Eukaryotic cell lines
☒	☐	Palaeontology and archaeology
☐	☒	Animals and other organisms
☒	☐	Clinical data
☒	☐	Dual use research of concern
☒	☐	Plants

Methods

n/a	Involved in the study	
☒	☐	ChIP-seq
☒	☐	Flow cytometry
☒	☐	MRI-based neuroimaging

Antibodies

Antibodies used	The primary antibodies: rabbit anti-G3BP1 (PA5-29455, Thermo Fisher Scientific); mouse anti-Tom20 (F-10, Santa Cruz); mouse anti-SPFQ (C23, MBL); mouse anti-SRSF1 (103, Invitrogen); rabbit anti-TDP-43 (80002-1-RR, Proteintech); mouse anti-SC35 (ab11826, Abcam); rabbit anti-SP100 (HPA016707, Sigma-Aldrich); mouse anti-NPM1 (FC82291, Sigma-Aldrich); rabbit anti-HP1alpha (2616S, Cell Signaling); mouse anti-Neurofilament H (SMI-32, Millipore); mouse anti-beta3 Tubulin (T5076, Sigma-Aldrich); rabbit anti-SFPQ (ARP4-572, aviva systems biology), mouse anti-alpha-Tubulin (DM1a, Sigma-Aldrich), mouse anti-puromycin (12D10, Merk Millipore), and mouse anti-GAPDH (G8795, Sigma); goat anti-HRP-Cy3 (123-165-021, Jackson Immunoresearch). The secondary antibodies: Alexa Fluor 488-conjugated anti-mouse, Alexa Fluor 594-conjugated anti-rabbit, anti-mouse, and Alexa Fluor 647-conjugated anti-rabbit and anti-mouse (Thermo Fisher Scientific); IRDye 800CW and IRDye680RD (LI-COR).
Validation	The signal or blotting patterns of the following antibodies were consistent to those in the corresponding previous studis: anti-G3BP1 and anti-puromycin, Guillén-Boixet et al, Cell 2020; anti- Neurofilament H, Bellmann et al, Biomaterials, 2019; anti-HRP, Mosca et al, 2012, Nature. The signal or blotting patterns of the following antibodies were consistent to those in the coresponding manufacture's websites: anti-Tom20, https://www.scbt.com/p/tom20-antibody-f-10?srsltid=AfmBOorEWaFi-ypmbeYqUt-dVwzVFOxiAh-oLi0f1fGPPK-bn9npHOwt; anti-TDP-43, https://www.ptglab.com/products/TDP-43-Antibody-80002-1-RR.htm); anti-SC35, https://www.abcam.com/en-us/products/primary-antibodies/sc35-antibody-sc-35-nuclear-speckle-marker-ab11826; anti-SP100, https://www.sigmaaldrich.com/US/en/product/sigma/hpa016707?srsltid=AfmBOopz-E2BlzbI55FqyvJd6n0ZpHz_JCAU_rvmptKv7aC26q87RyEZ; anti-NPM1, https://www.sigmaaldrich.com/US/en/product/sigma/b0556; anti-HP1alpha, https://www.cellsignal.com/products/primary-antibodies/hp1a-antibody/2616; anti-beta3 Tubulin, https://www.sigmaaldrich.com/US/en/product/sigma/t5076; anti-alpha Tubulin, https://www.sigmaaldrich.com/US/en/product/sigma/t6199; anti-GAPDH, https://www.sigmaaldrich.com/US/en/product/sigma/g8795. In addition to any manufacturer validation, SFPQ and SRSF1 antibodies were identified as most important for our assays and re-validated by Western blotting of an RNAi cell line, confirming loss of the specific band of expected molecular weight.

Eukaryotic cell lines

Policy information about cell lines and Sex and Gender in Research

Cell line source(s)	HeLa cell lines were FUS (MCB_005340), COIL (MCB_0002582), DCP1A (MCB_0003876), EWSR1 (MCB_0008863), PABPC1 (MCB_0006901), TIAL1 (MCB_0008967), and TRP53BP1 (MCB_0003740) from the database described in Poser et al. 2008 (DOI: 10.1038/nmeth.1199). iPS cell lines (KOLF, KOLF-C1, and AH-ALS1-F58) with FUS mutations were previously generated and described in Naumann et al 2018 (DOI: 10.1038/s41467-017-02299-1). WTC-11 iPS cells were from the Coriell Institute (GM25256). U2OS cells were from ATCC.
Authentication	Authentication procedures for iPS cell lines except for WTC-11 are described in their corresponding references. GM25256 and HeLa lines were not further authenticated, but each HeLa line was validated based on its signals of each GFP-tagged target protein.
Mycoplasma contamination	Routine DNA staining is used to identify any potential mycoplasma contamination. No samples had detectable mycoplasma.
Commonly misidentified lines (See ICLAC register)	We only used kyoto HeLa among various HeLa cell lines available in the research comminity as the parental HeLa cell line used in this study.

Animals and other research organisms

Policy information about studies involving animals; ARRIVE guidelines recommended for reporting animal research, and Sex and Gender in Research

Laboratory animals	The following C. elegans stains (derived from N2 strain) were used in this study: uqIs24[pmyo- 2::tagrfp::pab1gene]; uqIs9[pmyo-2::rho-1::tagrfp+ptph-1::gfp]. They were examined 48 h after switching the L4s from 20°C to 25°C (day 2 of adulthood). D. melanogaster strains used in this study were either from Bloomington Drosophila Stock Center (w1118, UAS-eGFP, D42-GAL4, and OK6-Gal4) or derived as described in Lanson et al 2011 (UAS-FUS WT and UAS-FUS R521C; DOI: 10.1093/hmg/ddr150), Anderson et al. 2018 (UAS-FUS P525L; DOI: 10.1242/jeb.179598) or Ritson et al. 2010 (UAS-TDP-43 WT and UAS-TDP-43 M337V; DOI: 10.1523/JNEUROSCI.5894-09.2010). They were examined at the adult stage within a few days after eclosion (for the climbing assay) or at the 3rd instar larval stage (for immunostaining).
Wild animals	None
Reporting on sex	No sex-based analysis, in line with standard practice for C. elegans and D. melanogaster.
Field-collected samples	None
Ethics oversight	Not applicable, non-cephalopod invertebrates.

Note that full information on the approval of the study protocol must also be provided in the manuscript.

Plants

Seed stocks	None
Novel plant genotypes	None
Authentication	None
