# Supplementary figures and images for: Small-molecule dissolution of stress granules by redox modulation benefits ALS models
Source: Nat Chem Biol. 2025 May 14;21(10):1577–88. doi: 10.1038/s41589-025-01893-5 (PMC12463676; doi:10.1038/s41589-025-01893-5)

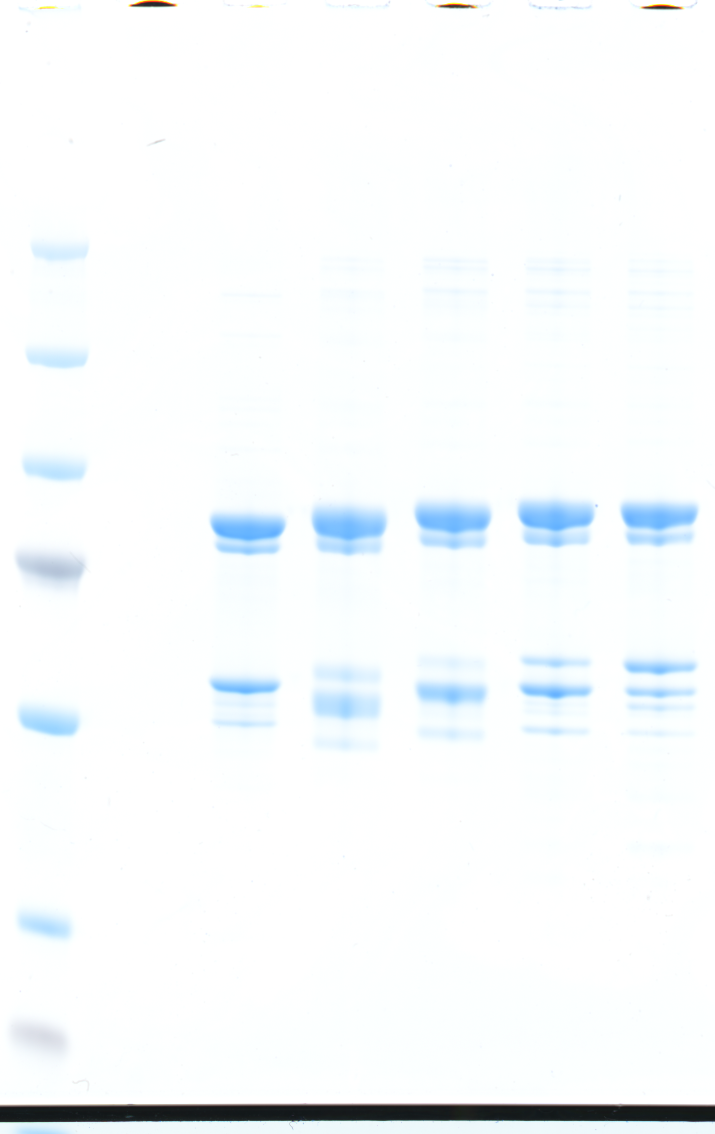

Supplement: Supplementary file 17 — Gel image. [file 41589_2025_1893_MOESM17_ESM.tif]
